# Spatial heterogeneity, trade-offs, and bundle identification of ecosystem services in karst watersheds: a comprehensive assessment of the Lijiang River Basin

**DOI:** 10.1038/s41598-026-49739-x

**Published:** 2026-04-27

**Authors:** Yali Feng, Yanling Huang, Zhongjun Wu, Ruize Sun, Yongkang Wang

**Affiliations:** 1 College of Environmental Science and Engineering, Guilin University of Technology, Guilin, 541006 China; 2 College of Tourism & Landscape Architecture, Guilin University of Technology, Guilin, 541006 China; 3 Guangxi Key Laboratory of Environmental Pollution Control Theory and Technology, Guilin University of Technology, Guilin, 541006 China; 4 University Engineering Research Center of Watershed Protection and Green Development, Guilin University of Technology, Guangxi Guilin, 541006 China; 5 Graduate School, Guilin University of Technology, Guilin, 541006 China

**Keywords:** Ecosystem services, Ecosystem service bundles, Lijiang River Basin, Trade-offs and synergies, Driving factors, Ecology, Ecology, Environmental sciences, Environmental social sciences

## Abstract

The Lijiang River Basin is under pressure from rapid urbanization and tourism. To support sustainable management, the present study assessed 10 ecosystem services (ESs) from 2000 to 2020 using the InVEST model and spatial statistics. Correlation analysis and a Self-Organizing Map neural network were combined to analyze ES interactions and identify service bundles, whereas Geodetector revealed driving factors. There was significant spatial differentiation of the services, with high-value areas for carbon sequestration, habitat quality, food production, and soil conservation in high-altitude forest regions, whereas low-altitude regions showed high water yield, recreational services, and nutrient retention due to human activity. There were notable interactions among the services, with most regulating services exhibiting spatial synergies, particularly in mountainous regions, while water purification faced trade-offs with other services. Five typical service bundles were identified, reflecting the interplay of natural resilience and human impact. Land use was the primary driver of service differentiation, with an increasing influence of socioeconomic factors. Based on the results, the study recommends differentiated governance strategies: strict protection of ecological supply zones and optimization of pressure response zones, which provide a scientific basis for ecological security and land planning in karst regions.

**Supplementary Information:**

The online version contains supplementary material available at 10.1038/s41598-026-49739-x.

## Introduction

Ecosystem services (ESs) are benefits that humans obtain directly and indirectly from ecosystems^1^. These include four categories: provisioning, regulating, cultural, and supporting services^2^, which serve as the material foundation and environmental guarantee for human production and life. Early understanding of ESs was limited to the material products they provide, neglecting the regulation, support, and cultural functions of ecosystems, which led to many environmental problems caused by blind development and expansion. The Millennium Ecosystem Assessment (MA) report showed that approximately 60% of global ecosystems have been in a state of decline over the past 50 years. The Intergovernmental Science-Policy Platform on Biodiversity and ESs indicated that 14 of the 18 natural capacities supporting human quality of life have declined over the past half-century^3^. These data reveal the severe trend of overall global ES degradation, posing challenges to achieving sustainable development goals. Therefore, the assessment, management, and utilization of ESs have become major issues facing regional development^4^, attracting widespread attention within academia.

Current quantification of ESs mainly employs two types of methods: monetization and non-monetization assessment. Monetization assessment, as exemplified by Costanza’s work^5,6^, advocates measuring regional ecology in monetary form to ultimately obtain ES values^7,8^. A Chinese scholar developed an equivalent factor table applicable to the Chinese context based on this approach^2,9^. Numerous scholars have applied this method to study regions such as Sichuan Province^10^, the Red River Hani Terrace Heritage Area^11^, Dianchi Lake^12^, the Yangtze River Delta urban agglomeration^13^, and the Southern Anhui International Tourism and Leisure Zone^14^. However, monetization methods struggle to reveal the intrinsic mechanisms and spatial processes of ES formation^15,16^, limiting their applicability in spatial precision governance. Therefore, internationally, there is a preference for a non-monetization assessment approach—the physical quantity method for research in this field. Physical quantity assessment is not affected by market prices and can reflect the spatial heterogeneity of services. Particularly, the InVEST model tool can directly quantify service physical quantities and reveal their spatial distribution, thus possessing significant advantages^17^. Many scholars have used the physical quantity method to select ES items for quantification and spatial visualization^18–20^. With the continuous improvement of this method, related research has gradually expanded from single service quantification to multidimensional systematic analysis, including hot and cold spot identification based on spatial autocorrelation^21^, trade-off/synergy relationship analysis between services^22–25^, service bundle clustering analysis^26–29^, and geographically weighted detector-driven factor detection^30^ at multiple levels. However, existing research still has obvious limitations: on the one hand, most achievements focus on only a few types of services, failing to comprehensively cover provisioning, regulating, supporting, and cultural services, with a particularly weak quantitative assessment of cultural services. Moreover, although some studies attempt multiple analytical methods, they often focus on only one aspect, lacking systematic research integrating “pattern-interaction-bundle-driver” as a whole, limiting the ability to understand ES complexity holistically.

As the location of the first group of national key scenic areas and a World Natural Heritage Site, the Lijiang River Basin possesses both prominent ecological functions and high ecological vulnerability, facing ecological pressures from tourism development and urbanization. This research adopting a multi-method integrated approach to systematically analyze ES spatial correlations, service bundle characteristics, and driving mechanisms remains insufficient. Given this context, this study aims to address a core scientific question: In a karst watershed undergoing rapid urbanization and tourism development, what are the spatial patterns of its multiple ecosystem services? How do these services interact with one another? And how do natural and anthropogenic factors drive the dynamic evolution of these patterns and relationships? To answer these questions, Based on a physical quantity assessment framework, this study aims to achieve the following objectives: (1) Clarify the spatiotemporal differentiation patterns of 10 ESs in the Lijiang River Basin from 2000 to 2020, including biodiversity conservation (BC), habitat quality (HQ), food production (FP), water conservation (WC), soil conservation (SC), carbon sequestration (CS), water yield (WY), recreational services (RS), nitrogen retention (NR), and phosphorus retention (PR); (2) Through hot and cold spot analysis and trade-off synergy identification, reveal the spatial clustering and interaction relationships among ESs; (3) Identify typical ES bundles and analyze their spatiotemporal evolution patterns; (4) Quantify the driving effects of natural and socioeconomic factors on services and service bundle patterns. This research aims to provide a scientific basis for ecological security pattern construction and land space optimization in the Lijiang River Basin.

## Study area and methods

### Study area

**Fig. 1:**
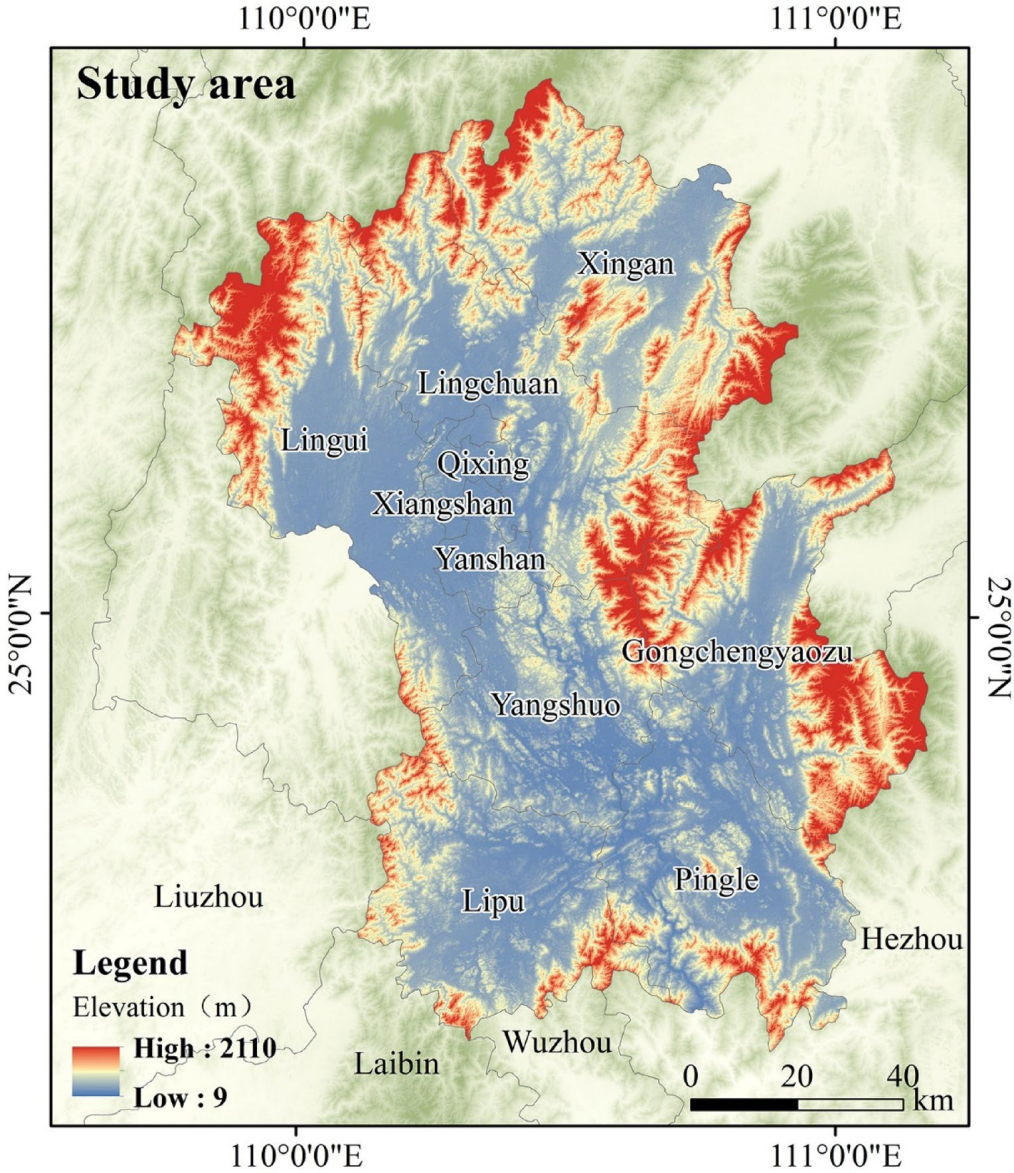
Map of the study area. This figure was generated from DEM data of the Geospatial Data Cloud platform (http://www.gscloud.cn). The study area boundary was extracted and delineated from the 1:1,000,000 public version of fundamental geographic information data released by the National Platform for Common Geospatial Information Services “Tianditu” (https://cloudcenter.tianditu.gov.cn) using ArcGIS 10.8 (http://www.esri.com/software/arcgis). This figure was created independently by the authors.

The Lijiang River Basin is located in northeastern Guangxi Zhuang Autonomous Region, China, with the river flowing through Guilin City (Fig. 1). The Li River originates from Maoer Mountain in Yuecheng Ridge, Xing’an County, Guilin City, and extends to the Sanjiangkou confluence in Pingle, as part of the Pearl River system. This study comprehensively considers the integrity of natural watersheds and the accessibility of socioeconomic data, combining hydrological boundaries with administrative divisions to determine the study scope (109°45’–111°10’E, 24°15’–25°54’N), covering 12 districts and counties, with a total area of 14,653.28 km^2^. The study area is surrounded by forest-covered, high-altitude mountains, with Yuecheng Ridge to the north, Tianping Mountain and Jiaqiao Ridge to the west, the remaining veins of Dayao Mountain on the southern edge, and Haiyang Mountain and Dupang Ridge running northeast–southwest to the east. The central-western region has a lower altitude, a dense population, and represents a typical karst landscape, which is also the location of the Li River Scenic Area. The study area belongs to a subtropical monsoon climate, with an average annual temperature of 18.8 °C, average annual evaporation of 1,482.5 mm, and average annual precipitation of 1,725.5 mm. As a world-class tourism destination, Guilin City experienced an increase in tourist number, rising from 15.8647 million in 2000 to 156.6366 million in 2024, with tourism revenue accounting for 76.58% of GDP in 2024, and the tertiary industry accounting for 55.3% of the overall industrial structure. This unique natural–socioeconomic composite background makes the Lijiang River Basin an ideal area for studying the interaction between human activities and ESs.

### Data sources

This study uses multi-source data, including remote sensing images, land use, and socioeconomic data, to determine ESs (Table 1). All data were unified to the WGS_1984_UTM_Zone_49N coordinate system, and raster data were resampled to a 30-m resolution, and the study area boundary was used as a unified range for clipping, processing, and analysis.

**Table 1:** Description of data source.

Data	Data source	Data type	Resolution
Land use/cover	Resource and Environment Science and Data Center (https://www.resdc.cn/) ^31^	Raster	30 m
Soil data	National Cryosphere Desert Data Center. (http://www.ncdc.ac.cn) ^32,33^	Raster	1 km
DEM	Geospatial Data Cloud (https://www.gscloud.cn/)	Raster	30 m
NDVI	National Ecosystem Science Data Center, National Science & Technology Infrastructure of China. (http://www.nesdc.org.cn) ^34,35^	Raster	30 m
Nighttime lights index	National EaRSh System Science Data Center(https://www.geodata.cn) ^36^	Raster	500 m
Population density	Resource and Environment Science and Data Center (https://www.resdc.cn/) ^37^	Raster	1 km
GDP density	Resource and Environment Science and Data Center (https://www.resdc.cn/) ^37^	Raster	1 km
Socio-economic data	The Guilin Statistical Yearbook (GSY)	TXT	\
Meteorological data	National EaRSh System Science Data Center(https://www.geodata.cn) ^38^	Raster	1 km
Basic geographic information data	National Catalogue Service For Geographic Information (https://www.webmap.cn/)	shp	\
National A-Grade Scenic Area Dataset	Ministry Of Culture And Tourism Of The People’s Republic Of China	shp	\
Depth to bedrock (DTB)	http://globalchange.bnu.edu.cn/research/cdtb.jsp ^39^	Raster	1 km
NPP	The MOD17A3H Product From NASA’s MODIS	Raster	500 m

### Research methods

#### Research technical route

**Fig. 2:**
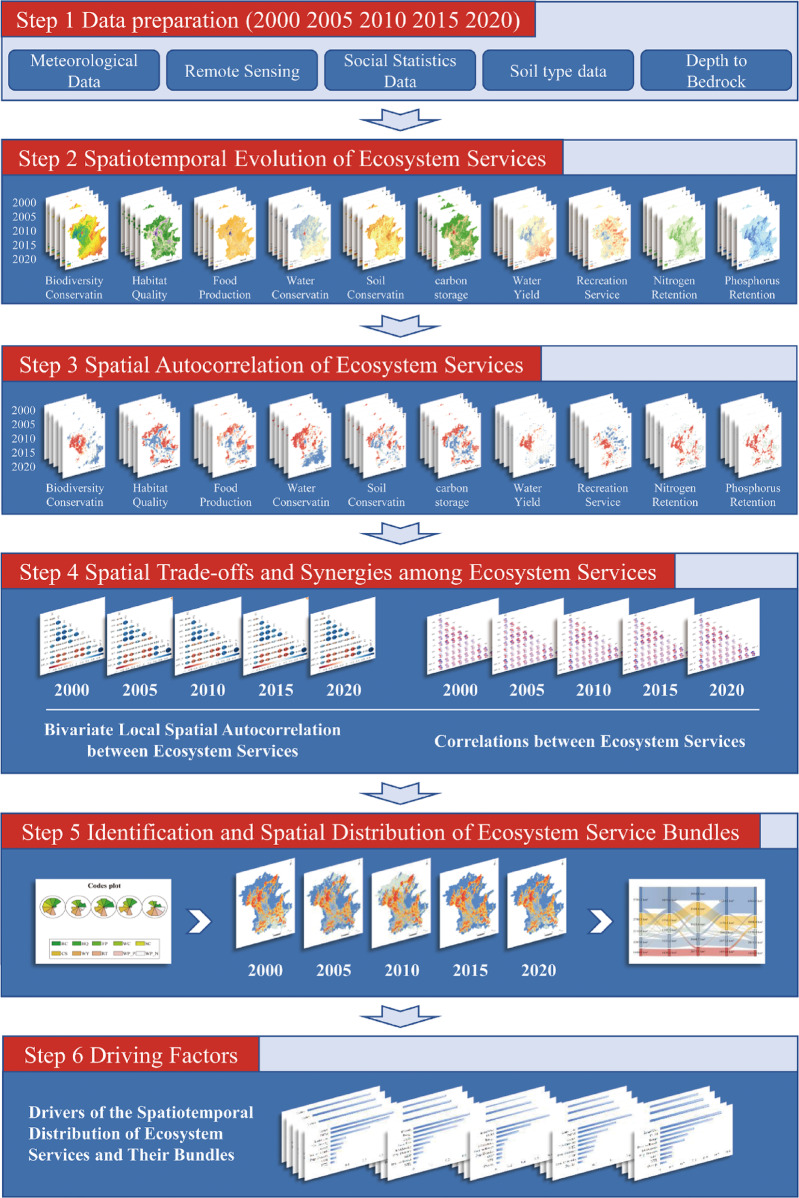
Technical flowchart.

Starting from ES supply, this study evaluated 10 ESs, visualized their spatial patterns, analyzed their spatial clustering and interrelationships, identified ES bundles, and explored the driving factors underlying their spatiotemporal evolution. The aim is to provide a scientific basis for the construction of ecological security patterns in the Lijiang River Basin (Fig. 2).

#### Spatiotemporal pattern evolution analysis of ESs

Based on the MA framework and the ES classification system constructed by Xie et al. according to China’s actual situation, combined with the natural, social, and economic characteristics of the Lijiang River Basin, this study selected 10 ES types: FP, WY, NR, PR, SC, BC, HQ, WC, CS, and RS, covering four major categories: provisioning, regulating, supporting, and cultural services. Using the physical quantity assessment method and comprehensively employing the InVEST model (v3.16.1; The Natural Capital Project, Stanford, CA, USA), ArcGIS (v10.8; Esri, Redlands, CA, USA), RStudio (v4.5.1; Posit Software, PBC, Boston, MA, USA), and Geoda (GeoDa Center for Geospatial Analysis and Computation, Tempe, AZ, USA)., quantification and spatial mapping of ESs were completed for five observation years (2000, 2005, 2010, 2015, and 2020) spanning a 20-year time series.

The assessment methods for each service are as follows (detailed assessment methods are provided in the Supplementary Material):

1. Biodiversity conservation: Integrating DEM, annual precipitation, annual average temperature, and net primary productivity (NPP) data, normalized using the range method, and calculated using the ArcGIS raster calculator according to the following formula (Esri, Redlands, CA, USA).
  1 $$S_{{bio}} = NPP_{{mean}} \times F_{{pre}} \times F_{{tem}} \times \left( {1 - F_{{ait}} } \right)$$
  Where: S_bio_ is the biodiversity maintenance service capacity index; NPP_mean_ is the mean annual net primary productivity; F_pre_ is the mean annual precipitation factor; F_tem_ is the mean annual temperature factor; F_alt_ is the altitude factor.
2. Habitat quality: Based on land use and road distribution data, threat factor tables and sensitivity tables were developed and applied through the InVEST HQ module.
3. Food production: The " Food production " assessed in this study represents an evaluation of integrated supporting potential. Beyond staple crop output from croplands, it incorporates livestock products dependent on forestland and grassland, as well as aquatic products from water bodies, using socio-economic data. The production capacity of cropland, forestland, grassland, and water bodies was spatially weighted and allocated based on the NDVI index. All product types were uniformly converted into standard grain yield equivalents to achieve the spatial visualization of food production.
4. Water conservation: Based on WY, combined with surface runoff coefficient correction, the difference between WY and surface runoff was used as the WC amount.
5. Soil conservation: SC was assessed using the InVEST model sediment delivery ratio module, based on the digital elevation model (DEM), soil, and other relevant data.
6. Carbon sequestration: Based on land use data, carbon pool values for each land type in the Lijiang River Basin were determined by consulting relevant literature. The total carbon storage of aboveground, belowground, soil, and dead organic matter carbon pools for various land types was calculated using the InVEST model carbon-storage and sequestration module.
7. Water yield: Based on annual precipitation, annual actual evapotranspiration, bedrock depth, and land use data, WY was determined using the InVEST WY model.
8. Recreational services: Using the Recreation Opportunity Spectrum model to construct an evaluation system from four dimensions: naturalness, accessibility, scenic spot density, and population density, the entropy weight method and analytic hierarchy process were used to determine indicator weights, achieving spatial quantification of RS through raster overlay.
9. Water purification (nitrogen, phosphorus): The InVEST nutrient delivery ratio module was used to assess the retention capacity of nitrogen and phosphorus elements. This study used the difference between load and export as the water purification service quantity.

#### ES correlation analysis

##### Hot and cold spot identification of ESs

Spatial autocorrelation analysis was employed. First, the Global Moran’s I index was calculated for each ES based on 1-km^2^ grid units to assess overall spatial clustering. Subsequently, hot spot analysis using the Getis-Ord Gi* statistic was conducted. Significant (*p* < 0.05) hot spots (clusters of high values) and coldspots (clusters of low values) were identified based on the z-score of each unit.

##### Trade-off and synergy analysis among ESs

To clarify the interactions among ESs, Spearman’s rank correlation coefficient was first used to analyze the relationships between service pairs: a positive value indicates synergy, whereas a negative value denotes a trade-off^40^. The absolute values of the correlation coefficients were interpreted as follows to indicate their strength: negligible (0.00–0.19), weak (0.20–0.39), moderate (0.40–0.59), strong (0.60–0.79), or very strong (0.80–1.00)^41^.

To reveal the spatial heterogeneity of the relationships, bivariate local spatial autocorrelation analysis was further performed using Geoda software. Here, “High-High” and “Low-Low” clusters represent spatial synergy, whereas “High-Low” and “Low-High” clusters indicate spatial trade-offs (detailed assessment methods are provided in the Supplementary Material).

#### ES bundle identification

ES bundles were identified using a Self-Organizing Map (SOM) neural network in RStudio (R v4.5.1, R Foundation for Statistical Computing, Vienna, Austria). Using 2010 as the baseline period, the SOM model was trained with data from the 10 ESs, and the optimal number of bundles was determined to be five. To ensure comparability of results across multiple periods (2000, 2005, 2015, and 2020), data from all years were standardized using the mean and standard deviation from the 2010 baseline before being mapped onto the trained SOM model, thereby obtaining spatial distributions under a unified classification framework. Finally, the functional composition and spatial pattern of each bundle were illustrated using radar charts and spatial distribution maps^42^. A Sankey diagram, generated in Origin (v2025; OriginLab Corp., Northampton, MA, USA), was used to visualize the area transfer and structural evolution among bundles over time. The detailed methodology for ES bundles identification is provided in the Supplementary Material.

#### Driving factor analysis

This study selected driving factors by integrating natural and socio-economic dimensions, with the selection grounded in ecological principles and the specific context of the study area.

Natural Driving Factors: Since temperature, humidity, and altitude are key factors causing spatial differentiation in vegetation and landscape, precipitation, air temperature, and elevation were included as independent variables.

Socio-economic Driving Factors: GDP, population density, and the Night-Time Light (NTL) index were selected as comprehensive indicators of regional economic development and human activity intensity. Road networks are a significant factor influencing landscape patterns, species migration, and the flow of ecosystem services. Scenic spot density is crucial within the Lijiang River Basin, where tourism is a pillar industry, serving as an important indicator of tourism development. Land use type represents the most direct outcome of human modification of the natural underlying surface. Consequently, GDP, scenic spot density, distance to roads, population density, land use type, and the NTL index were chosen as independent variables.

To identify the dominant factors driving spatial heterogeneity of ESs (and bundles), the Geodetector model was used to calculate the explanatory power (q-statistic) of each driving factor. The q-value is calculated as follows:

2 $$q = 1 - \frac{{\mathop \sum \nolimits_{{h = 1}}^{L} N_{h} \sigma _{h}^{2} }}{{N\sigma ^{2} }}$$

where h = 1, 2, …, L are grid cells;

Nh and $\:{\boldsymbol{\sigma\:}}_{\mathbf{h}}^{2}$ are the total number of sample cells and variance for layer h;

N and σ^2^ are the total number of cells and the sum of variances for the study area. The q value ranges from 0 to 1; the larger the value, the stronger the explanatory power of independent variable X on the dependent variable Y, and vice versa.

## Results

### Spatiotemporal patterns of ES supply

**Fig. 3:**
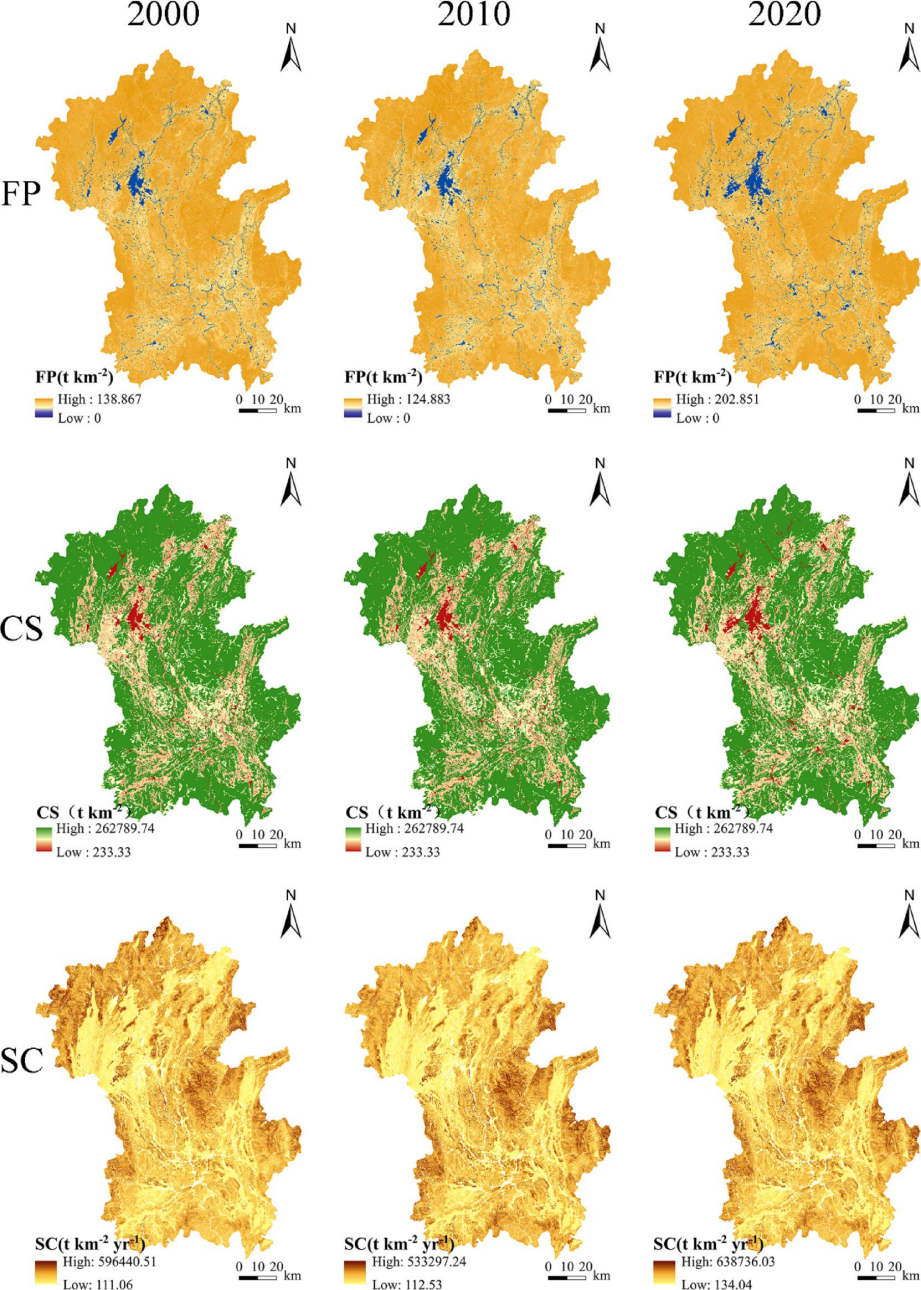

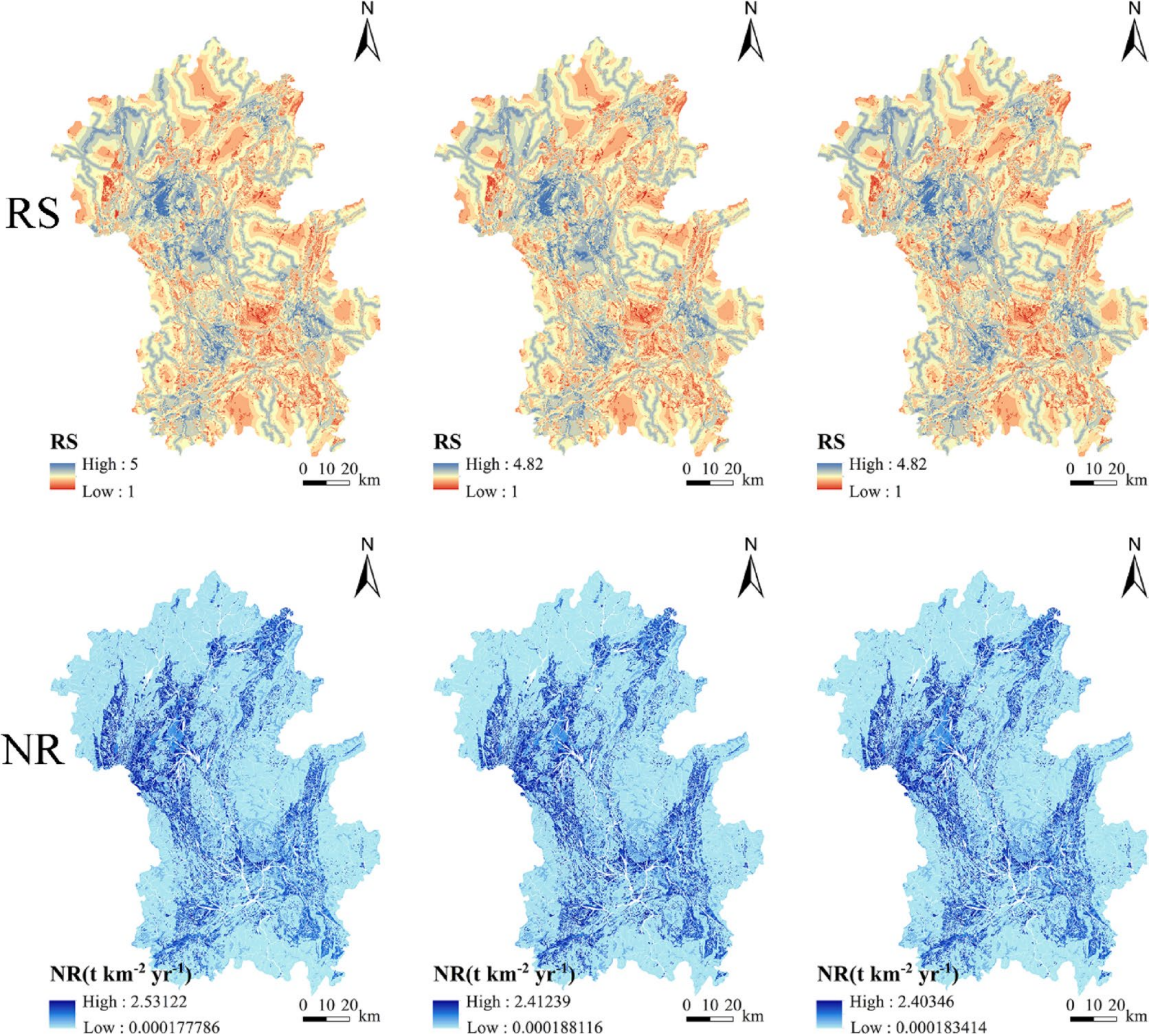
Spatiotemporal distribution of each ecosystem service. This figure presents the spatial patterns for three representative time points (2000, 2010, 2020) and five key services: Food Production, Soil Conservation, Carbon Sequestration, Recreational Services, and Nitrogen Retention. The complete spatial distribution maps for all 10 ecosystem services across all five observation years (2000, 2005, 2010, 2015, 2020) are available in the Supplementary Material (Fig. S1). In this figure, the subplots for SC, CS, and NR were generated using InVEST 3.16.1 (https://naturalcapitalproject.stanford.edu/software/invest) following the methods described in the “Research Methods” section, and were then processed using ArcGIS 10.8 (http://www.esri.com/software/arcgis). The subplots for other service types were generated using ArcGIS 10.8 (http://www.esri.com/software/arcgis) as described in the “Research Methods” section. All subplots were then composited into this figure using Adobe Photoshop 2020 (https://www.adobe.com/cn). This figure was created independently by the authors. FP, Food Production; CS, Carbon Sequestration; SC, Soil Conservation; RS, Recreational Services; NR, Nitrogen Retention.

From 2000 to 2020, ESs in the Lijiang River Basin exhibited a coexisting pattern of dynamic change and stability (Fig. 3, Fig. S1).

The high value of BC services slowly decreased from 0.677 in 2000 to 0.555 in 2020, while the mean value fluctuated upward from 0.186 in 2000 to 0.197 in 2020. From a spatial perspective, BC services overall exhibited a spatial pattern of high values in the central-western part and low values in surrounding mountainous areas, with high-value areas highly consistent with the inter-annual spatial pattern evolution of high-value areas for annual precipitation and NPP, showing a trend of first shrinking and then expanding, initially moving south and later north.

FP services, influenced by both NDVI and land use changes, showed a trend of initial growth followed by fluctuating decline, highly consistent with NDVI mean values and the areas of cultivated land, forest land, and grassland. From a spatial distribution perspective, low-value areas were mainly concentrated in urban–rural construction land and water bodies. These areas were primarily clustered within the six central districts of Guilin and the county seats, and they expanded annually with urban development. Areas with high values were predominantly distributed in high-altitude forestlands, a pattern that may appear counterintuitive. However, this is because the food production metric in this study primarily represents the “integrated supporting potential” of ecosystems for food production, rather than direct “crop yield.” We will elaborate on this point further in the Discussion section.

WC services are significantly affected by land use patterns and annual precipitation levels. Elevated WC values are observed in forested and grassland regions located in the northern and central-eastern mountainous areas of the study region. In contrast, medium WC values are found in the cultivated land areas of the central-western and southern sections, while low-value zones are identified within the six central districts and county seats of Guilin, exhibiting an overall increasing trend annually. Furthermore, the high- and low-value zones of WC demonstrate a distinct north-south oscillation over time, closely correlating with variations in annual precipitation. This evidence underscores the pivotal influence of both land use and climatic fluctuations on the WC service.

The mean values of CS and HQ services decreased slightly from 213457.79 t/km^2^ and 0.759 to 211789.01 t/km^2^ and 0.741, respectively, reflecting ecological pressure from construction land expansion. High-value areas for these two services were primarily concentrated in forest-covered areas, including northern Yuecheng Ridge, western Tianping Mountain, central-eastern Haiyang Mountain, and southern Dayao Mountain, with medium–low value areas mainly concentrated in cultivated land, grassland, water bodies, and construction land in the central-western part. Compared with the CS function, the low-value areas of HQ were more closely aligned with urban–rural construction land and transportation facility directions.

The high values of the WY service showed no significant differentiation over time, exhibiting a trend of first decreasing then increasing, whereas the mean and low values increased from 982.3 to 139.1 mm to 1,118.9 and 362.3 mm, respectively, showing a slow upward trend. From the perspective of spatial differentiation, low-value areas were mainly distributed in Yuecheng Ridge in the northern part of the study area, Haiyang Mountain in the east, and the remaining veins of Dayao Mountain in the southern end, oscillating north–south between mountainous areas following precipitation patterns. In contrast, high values were stably distributed in low-altitude cultivated and construction land in the central-western part.

High-value zones for RS were consistently located in Guilin’s main urban area, Yangshuo, and other scenic spot-intensive areas, extending northward with road network improvement and tourism development, demonstrating strong human influence.

For water purification services, high-value areas for NR and PR were stably distributed in agricultural and urban regions in the central-western part, indicating that these areas serve both as nitrogen and phosphorus sink zones and as high-load-bearing areas for these nutrients. High values for SC services were concentrated in steep-slope forested lands, including the northern Yuecheng Ridge and Tianping Mountain, the central-eastern Haiyang Mountain, the western Jiaqiao Mountain, and the southern Dayao Mountain remnants, with changes positively correlated with rainfall erosivity. The commonality between water purification and SC is that a high supply of these two ESs is driven by high demand.

### Hot and cold spot distribution of ESs

**Fig. 4:**
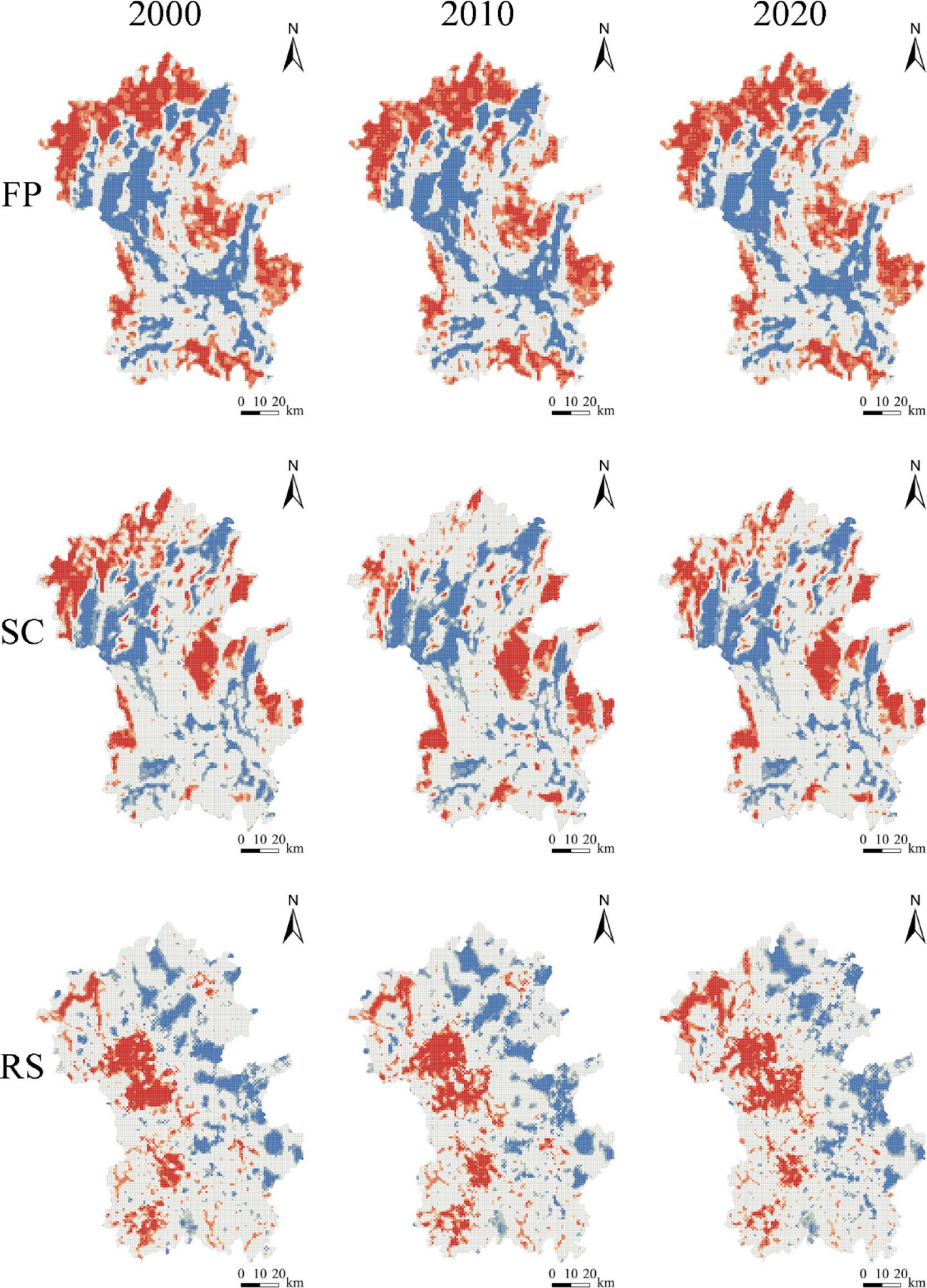

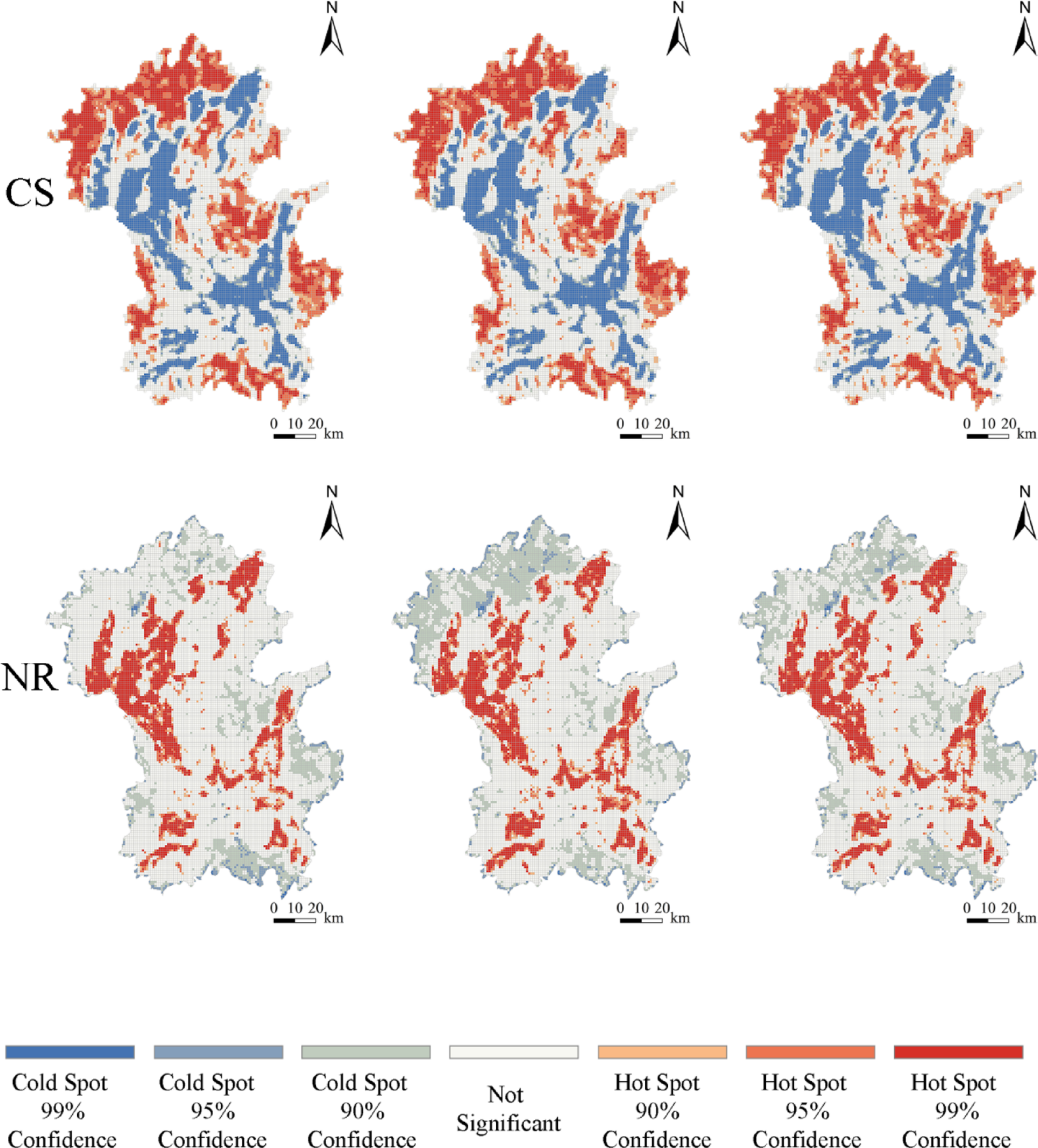
Hot and cold spot distribution of each ecosystem service. This figure presents the spatial patterns for three representative time points (2000, 2010, 2020) and five key services: Food Production, Soil Conservation, Carbon Sequestration, Recreational Services, and Nitrogen Retention. The complete spatial distribution maps for all 10 ecosystem services across all five observation years (2000, 2005, 2010, 2015, 2020) are available in the Supplementary Material (Fig. S2). The subplots in this figure were generated using ArcGIS 10.8 (http://www.esri.com/software/arcgis), and all subplots were then composited into this figure using Adobe Photoshop 2020 (https://www.adobe.com/cn). This figure was created independently by the authors. FP, Food Production; CS, Carbon Sequestration; SC, Soil Conservation; RS, Recreational Services; NR, Nitrogen Retention.

The global Moran’s I z-values for all ESs were positive and significant, indicating positive spatial autocorrelation, with high or low values tending toward spatial clustering. Hot spot analysis (Fig. 4, Fig. S2) showed that hot spots for WY, BC, NR, and PR services clustered in central-western low-altitude areas, while those for CS, HQ, SC, and FP were distributed in surrounding mountainous areas, forming complementary spatial patterns. WC and RS exhibited spatial differentiation characteristics, where cold and hot spot areas divided the study area into two, with WC showing a trend of north–south alternation in cold and hot spot distribution. Meanwhile, RS consistently maintained eastern hot and western cold characteristics during the observation period.

### Interactions among ESs

**Fig. 5:**
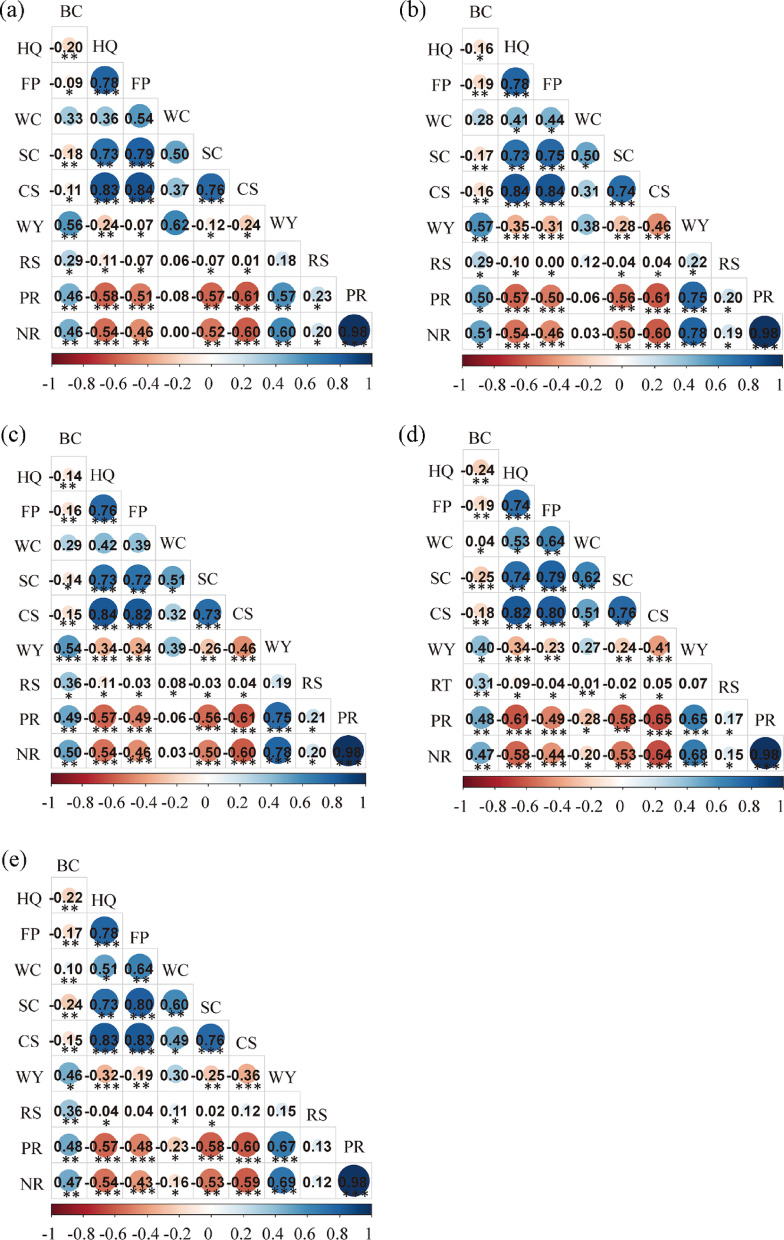
Heatmap of ecosystem service pair correlations. (**a**) 2000;(**b**) 2005;(**c**) 2010;(**d**) 2015;(**e**) 2020. The subplots in this figure were generated using R 4.3.1 (https://www.r-project.org/), and all subplots were then composited into this figure using Adobe Photoshop 2020 (https://www.adobe.com/cn). This figure was created independently by the authors. BC, Biodiversity Conservation; HQ, Habitat Quality; FP, Food Production; WC, Water Conservation; SC, Soil Conservation; CS, Carbon Sequestration; WY, Water Yield; RS, Recreational Services; NR, Nitrogen Retention; PR, Phosphorus Retention.

**Fig. 6:**
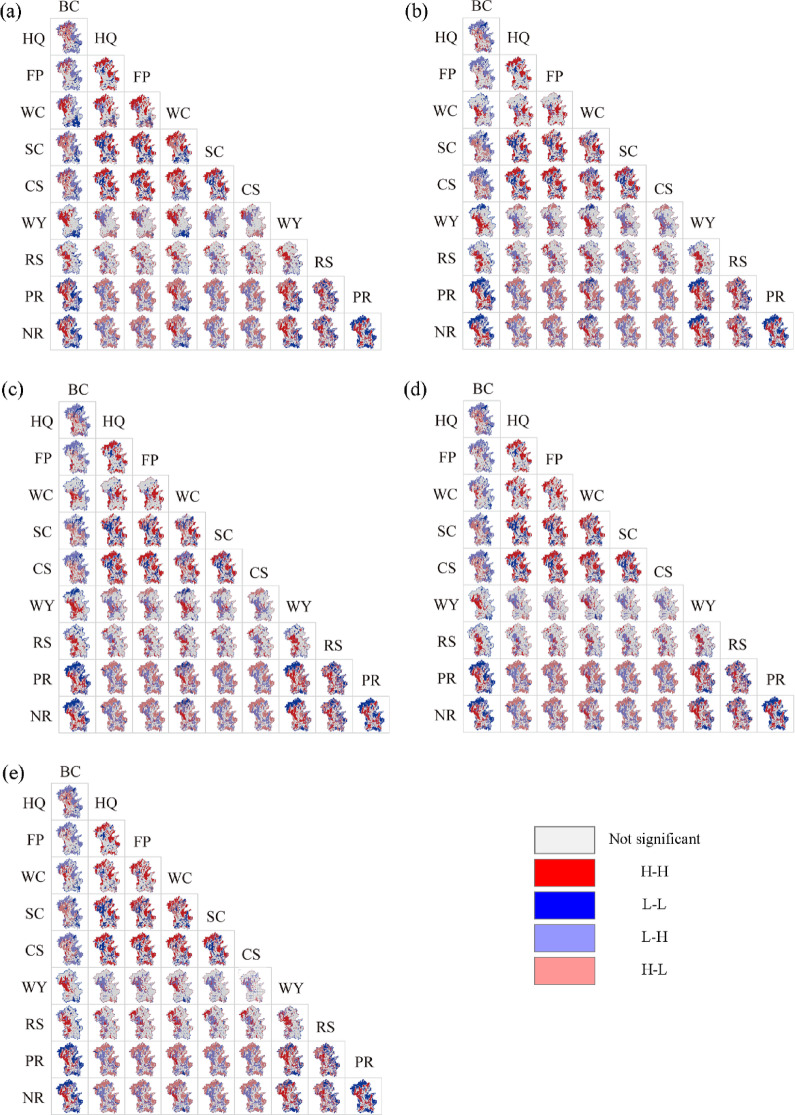
Spatial distribution of ecosystem service pair correlations. (**a**) 2000;(**b**) 2005;(**c**) 2010;(**d**) 2015;(**e**) 2020. The subplots in this figure were generated using GeoDa 1.22 (https://geodacenter.github.io/index-cn.html), and all subplots were then composited into this figure using Adobe Photoshop 2020 (https://www.adobe.com/cn). This figure was created independently by the authors. BC, Biodiversity Conservation; HQ, Habitat Quality; FP, Food Production; WC, Water Conservation; SC, Soil Conservation; CS, Carbon Sequestration; WY, Water Yield; RS, Recreational Services; NR, Nitrogen Retention; PR, Phosphorus Retention.

Only results with statistical significance (*p* < 0.05) and moderate or higher correlation strength were described. From the correlation heatmap (Fig. 5), as time progressed, the correlation coefficient signs between ES pairs, i.e., trade-off synergy relationships, showed no change. Correlation coefficients were positive for HQ-FP, HQ-WC, HQ-SC, HQ-CS, FP-SC, FP-CS, WC-SC, SC-CS, BC-WY, BC-PR, BC-NR, WY-PR, WY-NR, and NR-PR, with the spatial synergy proportion significantly higher than the trade-off proportion, showing significant synergistic relationships. The high-high clustering areas of most ES pairs were located in northern, central-eastern, and southern high-altitude areas, while low-low clustering was distributed in central-western low-altitude areas. However, it is worth noting that NR-PR exhibited opposite spatial distribution patterns. Correlation coefficients were negative for CS-WY, HQ-NR, HQ-PR, FP-NR, FP-PR, SC-NR, SC-PR, CS-NR, and CS-PR, with spatial trade-off proportions significantly higher than spatial synergy proportions, showing significant trade-off relationships (Fig. 6).

### Identification and spatiotemporal evolution of ES bundles

**Fig. 7:**
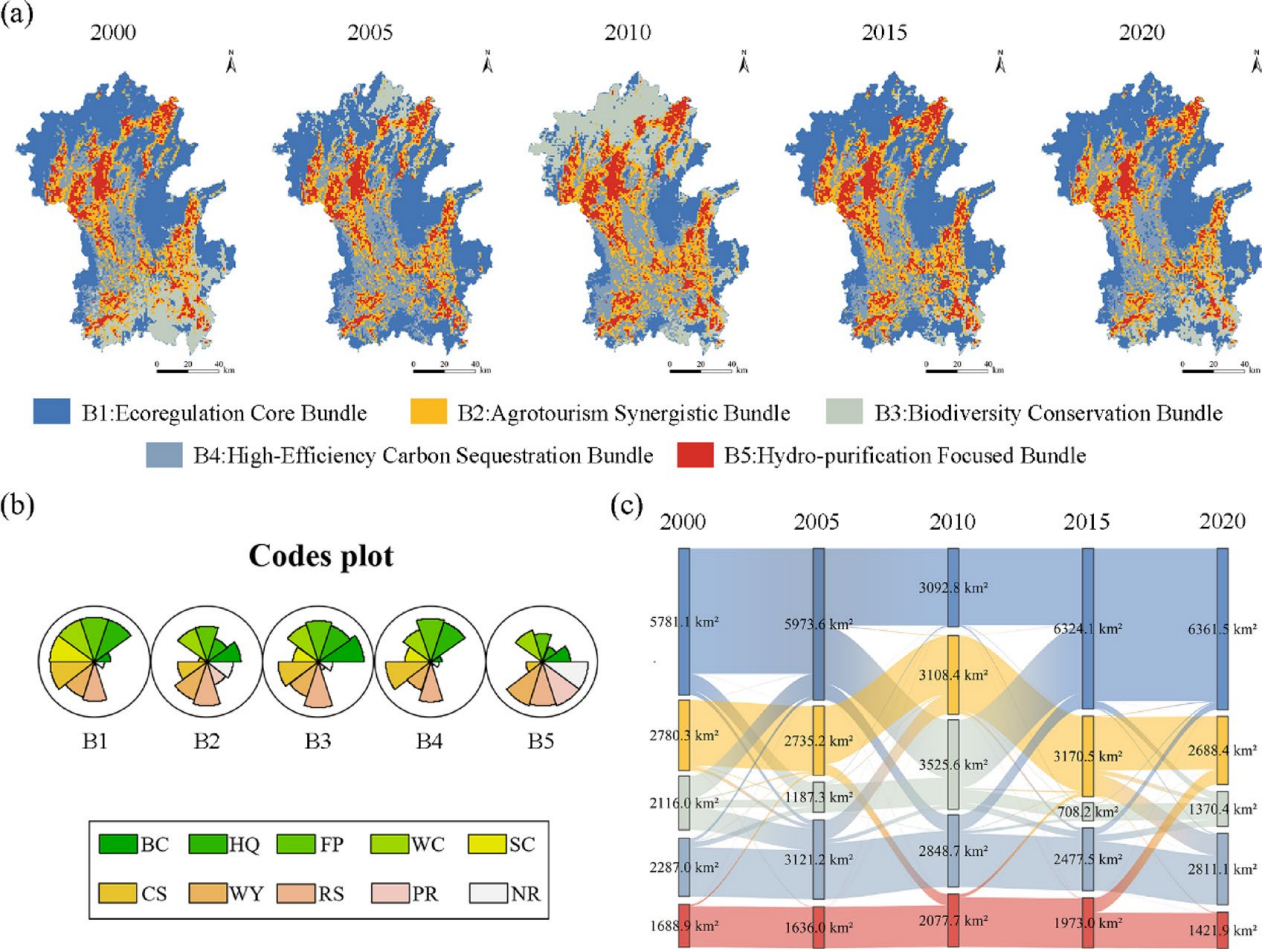
Ecosystem service bundle composition, distribution, and area changes. (**a**) Spatial-temporal patterns of ES bundles at the grid scale. (**b**) Composition and relative magnitude of ESs in ES bundles at the grid scale. Longer segments represent higher ES supply. (**c**) The area of interconversion among different ES bundles in 2000–2020 at the grid scale. In this figure, subplot (**a**) was generated using ArcGIS 10.8 (http://www.esri.com/software/arcgis) after data processing with R 4.3.1 (https://www.r-project.org/); subplot (**b**) was generated using R 4.3.1 (https://www.r-project.org/); subplot (**c**) was generated using Origin 2021 (https://www.originlab.com/ ). All subplots were then composited into this figure using Adobe Photoshop 2020 (https://www.adobe.com/cn). This figure was created independently by the authors. BC, Biodiversity Conservation; HQ, Habitat Quality; FP, Food Production; WC, Water Conservation; SC, Soil Conservation; CS, Carbon Sequestration; WY, Water Yield; RS, Recreational Services; NR, Nitrogen Retention; PR, Phosphorus Retention.

SOM clustering identified five typical service bundles (Fig. 7a, b). The first service bundle centers on HQ, WC, SC, FP, and CS functions, constituting a comprehensive ecological regulation system. WY and RS also performed well, but water purification services were weak. This bundle is concentrated in the central-eastern Haiyang Mountain and Dupang Ridge, the western edge of Jiaqiao Ridge, and the remnant areas of the southern edge of Dayao Mountain. These areas are forest land of higher altitude, possessing high ecological integrity and prominent environmental regulation functions, serving as important ecological barriers, and are thus named the Ecoregulation Core Bundle(ECB). The second service bundle is characterized by RS, while also maintaining high levels of biodiversity, FP, WC, and other functions, exhibiting typical multifunctional characteristics. This bundle is primarily found within low-altitude cultivated areas, forested lands, and grasslands in the central-western region. This distribution highlights the interplay and equilibrium among provisioning, cultural services, and ecological regulation functions influenced by human activity, and is thus named the Agrotourism Synergistic Bundle(ASB). The third service bundle is characterized by BC functions, encompassing HQ, FP, WC, and CS functions, serving as the study area’s “gene pool” for maintaining biodiversity. It was mainly distributed in forest land in northern Yuecheng Ridge and northern Tianping Mountain, scattered on the southern edge of Dayao Mountain remnant forest land, serving as an important regional “water tower,” with its core role being maintaining species survival and habitat security; it was designated the BC Bundle(BCB). The fourth service bundle was dominated by CS services, while FP and HQ also maintained high levels. It was mainly distributed in low-altitude forest land in the central-western and southwestern parts, serving as an important regional “carbon sink”; it was named the High-Efficiency CS Bundle(HCB). The fifth bundle was jointly driven by WY and nitrogen- and phosphorus-purification functions, with developed RS, but generally low natural ecological regulation functions (such as SC and CS). Its spatial distribution (cultivated land and construction land) and functional combination indicated that it is a highly artificial system serving human settlements, with core functions of ensuring water resources and absorbing pollutants generated by human activities; it was named the Hydro-purification Focused Bundle(HFB).

The Sankey diagram (Fig. 7b, c) shows that from 2000 to 2020, the spatiotemporal distribution pattern of ES bundles in the study area exhibited both strong stability and significant local functional transformation. Specifically, during the 2000–2005 stage, the Ecoregulation Core Bundle had the largest area, with nearly 5,000 km^2^ remaining unchanged in type, reflecting its resilience as the regional ecological foundation. The BC Bundle showed some degree of differentiation, with some areas (approximately 879 km^2^) converting to the Ecoregulation Core Bundle and gradually moving to northern forested mountainous areas of the study area, suggesting that its ecological function evolved toward comprehensive regulation. The Hydro-purification Focused and Agrotourism Synergistic bundles were most stable, showing their high coupling with human activities.

During 2005–2010, inter-bundle transformation became more active. Approximately 2,273 km^2^ of the Ecoregulation Core Bundle transformed into the BC Bundle. However, this did not last, as in the subsequent observation period, this trend reversed on a larger scale (2,688 km^2^), highlighting the high integration and dynamic relationship between BC and comprehensive regulation functions in ecosystems. Approximately 443 km^2^ of the Agrotourism Synergistic Bundle transformed into the Hydro-purification Focused Bundle, suggesting that further urbanization strengthened hydrological purification and recreational functions.

Between 2010 and 2015, the High-Efficiency CS Bundle exhibited significant durability, with minor components transitioning into the Ecoregulation Core Bundle. This transition suggests that a well-defined spatial pattern as a carbon sink functional zone was established, characterized by enhanced regulatory functions and a progressive improvement in ecological quality.

During 2015–2020, the Ecoregulation Core Bundle further integrated portions of the High-Efficiency CS Bundle areas, strengthening its position as the regional ecological core. Mutual transformation occurred between the Agrotourism Synergistic and High-Efficiency CS bundles, reflecting dynamic trade-offs among provisioning, recreational, and CS functions in mid-low-altitude areas. Although the Hydro-purification Focused Bundle remained generally stable, some areas (approximately 580 km^2^) transformed into the Agrotourism Synergistic Bundle, potentially indicating local adjustments in ecological functions within human activity-intensive zones.

### Dominant driving factors of ESs and service bundles

As shown in Fig. S3, for ESs, land use type showed continuous and strong explanatory power in most services (*q*-values generally higher than 0.45), especially most prominent in CS, NR, PR, FP, WC, HQ, and SC services, indicating that land use structure is a core element affecting ecosystem functions. Topography (as represented by DEM) significantly influenced SC, HQ, CS, FP, and water purification services, reflecting topography’s control over material transport and ecological processes. Precipitation dominated the spatial pattern of WY services, reflecting the high dependence of hydrological processes on climate factors. Additionally, the explanatory power of socioeconomic factors (such as population density and nighttime light index) for RS and FP continued to strengthen, indicating that the influence of human activities on cultural services and some provisioning services has been deepening.

For ES bundles, temperature and topography (as represented by DEM) showed strong explanatory power in most years, while the influence of land use significantly increased after 2015, indicating that bundle formation is co-regulated by natural and anthropogenic factors, with human-dominated land use changes gradually emerging as an important driving force in recent years.

Overall, natural factors (such as precipitation and topography) play a dominant role in provisioning and regulating services, while the influence of socioeconomic factors (such as GDP, population density, and nighttime lighting) on cultural services and some provisioning services has strengthened annually, reflecting the profound impact of human activities on ES patterns.

## Discussion

### Pattern analysis of ES spatial differentiation: formation mechanisms of ecological supply and pressure response zones

An interesting phenomenon emerged in the research results: WY, BC, NR, and PR services were lower in high-altitude areas than in low-altitude areas, and lower in forest areas than in other land types. Similar findings exist in related research^43^. We attempt to explain this formation mechanism from two perspectives: local supply and pressure response.

High-altitude forest areas serve as “ecological supply zones,” primarily providing regulating services such as CS, HQ, and SC. Notably, the spatial distribution pattern of SC services revealed in this study and its relationship with vegetation cover types differ slightly from those in some existing research^44^, while the distribution patterns of other regulating services are consistent with those from previous research conclusions^45^. The sustained high values of these services may stem from the inherent structural characteristics of forest ecosystems, such as higher vegetation carbon density, complex community structure, and environmental conditions far from human disturbance^46^. The FP service assessed in this study focuses on the total supporting capacity of ecosystems. This includes not only the direct output of grain and cash crops from croplands, but also livestock products dependent on fodder supplied by forest and grassland ecosystems, as well as aquatic products derived from water bodies. Therefore, the high values observed in mountainous areas indicate that these ecosystem units hold high supportive value within the watershed’s food supply system, reflecting the potential importance of these regions as sources of feed for livestock farming.

In contrast, low-altitude areas exhibit obvious “pressure response type” service characteristics. The high values of WY services are influenced not only by the spatial distribution of annual precipitation but also by lower evapotranspiration water consumption from cultivated land and construction land. The high values of BC services reflect the high primary productivity of this area under the combined effects of favorable hydrothermal conditions and human management, as well as the protective value of natural patches in mitigating human disturbance^47^. What is instructive is the high value of water purification services, which essentially results from the combined effects of higher exogenous nutrient loads (from agricultural and domestic wastewater) in human activity-intensive areas and ecosystem retention capacity, characterizing total purification “quantity” rather than purification efficiency. Additionally, the central-western part of the study area is where the central urban area of Guilin and the core section of the Li River Scenic Area are located, with dense road networks, concentrated scenic spots, and high population density. The spatial distribution pattern of RS, showing high values in the west and low values in the east, is consistent with actual conditions and data characteristics.

The aforementioned spatial heterogeneity reflects the coupling effect of the “natural topographic gradient” and the “human activity intensity gradient.” High-altitude forest areas experience minimal anthropogenic disturbance and maintain well-preserved vegetation cover, allowing the natural regulatory functions of ecosystems—such as CS, SC, WC—to be fully realized. In contrast, low-elevation zones are characterized by concentrated population and socio-economic activities. This is particularly evident in the Lijiang River Basin, where tourism serves as the dominant and pillar industry. The distinctive characteristics of its economic activities lead to a high spatiotemporal concentration of human presence. Consequently, intensive demands for water use, domestic wastewater discharge, and tourism infrastructure development drive correspondingly high values for WY, NR, PR and RS in these areas.

Based on these findings, we can distill the formative mechanisms underlying the spatial heterogeneity of ecosystem services in the Lijiang River Basin: the natural background (topography, vegetation) governs the fundamental pattern of ecosystem services, while human activity pressure (threat sources, tourism infrastructure) and climatic fluctuations respectively shape the distinct spatial patterns of specific services, such as water yield and water purification. Compared with conclusions from studies on karst forests in Guizhou, which identified climate as the primary driver of spatial variation^48^, this study highlights more prominently the “shaping” role of human activity pressure on the spatial configuration of specific services in the Lijiang River Basin under conditions of high-intensity tourism disturbance.

### Spatial patterns and formation mechanisms of ES trade-offs and synergies

The trade-off and synergy relationships among ecosystem services exhibited distinct spatial patterns. Regulating services (WC, SC, CS) all depend on specific topography, soil structure, and favorable vegetation conditions. Consequently, they demonstrated high-high clustering characteristics in high-altitude forestlands with favorable natural conditions, forming a synergistic core area for ecological regulation. In contrast, water purification services (PR, NR) are closely associated with human agricultural activities (e.g., fertilizer application) and tourism activities (e.g., domestic sewage discharge). Therefore, their high-value areas significantly overlapped with agricultural land and urban construction land, showing a clear trade-off relationship with the regulating services. This reveals an inherent spatial conflict between water purification functions and other ecological functions, driven by external nutrient loads from human activities. This study further elucidates that human activities, particularly tourism, profoundly shape the spatial patterns of trade-offs and synergies among services by altering land use and increasing external nutrient loads^49^.

### ES bundle dynamics reveal system resilience and evolution of human–environment relationships

The spatial pattern of ES bundles is likewise a direct reflection of ecological background and human activities. The reason the Ecoregulation Core and BC bundles are distributed in high-altitude mountainous areas is that they jointly depend on well-preserved natural forest ecosystems. These areas have little human disturbance, complex vegetation structure, and large biomass, enabling them to stably and efficiently providing multiple regulating and supporting services, thereby constituting the basic framework of regional ecological security. We do not consider their mutual transformation as functional degradation; it is more likely to reflect the dynamic balance and integrated synergy exhibited by forest ecosystems under natural succession or a climate change background, as well as in micro-habitats with different altitudes and aspects, thereby demonstrating the system resilience of the natural background.

The spatial distribution of service bundles in mid-low-altitude areas is deeply influenced by human activities, resulting from human–environment coupling. The stable existence of the Agrotourism Synergistic Bundle demonstrates the possibility of simultaneously achieving agricultural production, cultural services, and basic ecological functions in semi-natural landscapes. The distribution of the Hydro-purification Focused Bundle indicates that urbanization advancement has gradually shifted the dominant functions of semi-natural landscapes toward pollution absorption, reflecting that the demand by human activities for specific services (such as water purification and RS) inevitably leads to the decline of other natural regulating services, presenting typical trade-off characteristics^50^. The transformation of this service bundle to the Agrotourism Synergistic Bundle reflects responses to ecological pressure; ecological restoration measures have yielded initial results, with ecological functions showing local optimization and recovery. The mutual transformation between the HCB and the ASB, with 2010 as a critical temporal node, was likely influenced by the dual effects of urban land expansion and the Grain for Green policy^51,52^. Analysis of driving factors using the Geodetector indicated that land use type consistently remained the dominant driver, while the influence of socio-economic factors such as the Night-Time Light (NTL) index and population density exhibited a continuous increase. These findings fully reveal the compound process through which both natural and anthropogenic factors have reshaped the landscape patterns.

### Dual driving mechanisms of the natural environment and human activities

The driving mechanisms of ecosystem services in the Lijiang River Basin, as revealed by this study, can be synthesized into a fundamental framework of “natural geographic gradients establishing the baseline, overlaid and reshaped by human activities.”

First, the driving effects of individual factors on specific services corroborate their inherent ecological relationships. Land use type, by altering vegetation structure and underlying surface properties, directly regulates the generation and transfer of provisioning, supporting, and regulating services. Serving as the dominant driver for most services and the foundation for their generation, this finding further validates previous research^18,53,54^. The strong explanatory power of DEM for services such as SC, HQ, FP, and CS originates from its control over regional hydrothermal conditions, soil erosion dynamics, and human accessibility. This fundamentally delineates ecological regulation zones from areas of human disturbance, establishing the basic pattern of spatiotemporal heterogeneity in ecosystem services. Precipitation and temperature primarily influence services like WY and WC by driving regional hydrological cycles and vegetation physiological processes^55,56^. This aligns with the fundamental principle that hydrothermal conditions govern eco-geographical differentiation, a relationship supported by relevant studies^57^.

Second, the dynamic evolution of driving forces reveals trends in human-land relationships. From 2000 to 2020, the regional GDP of Guilin increased more than sevenfold. Tourist arrivals grew nearly tenfold between 2000 and 2024, with tourism revenue accounting for 76.58% of the GDP in 2024. The increasing influence of socio-economic factors (e.g., population density, Night-Time Light index) not only represents intensified development but also drives rapidly growing demands for RS, WY, and FP. This indicates that human activities in the Lijiang River Basin have evolved from mere spatial occupation of ecological areas to a more complex reshaping of service provision, where rising demand stimulates increased supply—a trend corroborated by the high-value clustering of water purification functions. The consistently rising influence of land use on ES bundles similarly demonstrates that humans are integrating the combinatorial relationships of ecosystem services in the study area by altering land use patterns, a discovery that resonates with research on land management driving service synergies and trade-offs^58^. Compared to findings from karst forest studies in Guizhou, where climatic factors were prominent^48^, the significantly enhanced driving force of land use and socio-economic factors in this study confirms an evolution of the driving mechanism from “nature-dominated” to a “balanced natural-anthropogenic dual driving” under high-intensity human disturbance, especially from tourism. This represents a common characteristic of rapidly developing karst regions^49^.

### Ecological management implications

The ecosystem service bundles identified in this study provide clearly delineated units for precise “categorized and zoned” ecological management.

For high-altitude forestlands—where the ECB and the BCB are predominantly located—these areas serve as both the genetic reservoir for maintaining biodiversity and a crucial “water tower” within the watershed. It is recommended to incorporate these zones into ecological conservation redlines and enforce strict protection measures. Apart from appropriately conducted scientific monitoring and strictly controlled ecotourism with visitor caps, all types of developmental and construction activities should be prohibited. Vegetation restoration projects prioritizing natural recovery supplemented by human-assisted measures should be implemented, with focused protection of key ecological communities to preserve their core functions of high carbon sequestration and soil-water conservation.

The low-elevation regions experiencing intense human-land interaction are also focal points where the ASB, the HCB, and the HFB intersect. These areas must fulfill substantial provisioning services while simultaneously providing significant regulating services, making them hotspots for trade-offs and conflicts among various services. The core of management here lies in optimizing land-use structure, scientifically planning living, ecological, and production spaces, enhancing synergies among ecosystem services, and mitigating trade-off damages. Specifically, urban expansion should be strictly controlled, sufficient recreational space should be provided, and green infrastructure within built-up areas should be improved to alleviate urban heat-island, rain-island, and other multi island effects. In concentrated cropland areas, agricultural non-point source pollution control zones should be delineated, and ecological buffer strips should be promoted to reduce pollutant runoff into rivers and lakes through vegetative barriers and terrain regulation, thereby preventing algal blooms and protecting aquatic biodiversity.

In areas with high recreational service values, a balance between ecological protection and tourism development must be struck through a combined strategy of “smart reservation – zoned visitation – dynamic pricing.” This includes establishing a real-time tourist monitoring and early-warning system, optimizing tourist routes through ecological corridor identification to design diverse itineraries (e.g., hiking, cycling, water tours), and implementing reasonable seasonal price fluctuations for attractions to adjust peak-season visitor numbers, thereby alleviating ecological pressure.Ultimately, through the measures outlined above, the precise matching and sustainable enhancement of ecosystem service supply and demand can be achieved.

Methodologically, this study constructed an analytical framework of “spatiotemporal pattern visualization – correlation analysis – service bundle identification – driving force quantification.” It comprehensively applied spatial autocorrelation to reveal service aggregation patterns, used correlation analysis and bivariate spatial autocorrelation to clarify trade-offs/synergies and their spatial heterogeneity, introduced the SOM method to identify service bundles, and employed the Geodetector to quantitatively parse the driving contributions of natural and anthropogenic factors. Theoretically, based on the analytical results, a spatial pattern of “ecological provisioning zones – pressure-response zones” was delineated, and ecological management strategies were proposed according to the identified service bundles. Through the integration of methodology and theory, the study progressed from describing patterns to explaining mechanisms, thereby answering the initial scientific questions and providing directly applicable scientific evidence and a theoretical framework for the refined territorial spatial governance of the Lijiang River Basin and other similar regions.

## Conclusion

This study systematically assessed the spatiotemporal patterns, interaction relationships, and combinatorial characteristics of ESs in the Lijiang River Basin from 2000 to 2020, revealing their natural–socioeconomic driving mechanisms. This study showed that Watershed ESs exhibit clear spatial differentiation; high values for CS, HQ, FP, and SC are concentrated in peripheral high-altitude forest regions, forming “ecological supply zones,” while high values for WY, RS, and water purification are distributed in human activity-intensive low-altitude areas, manifesting as “pressure response zones.” Synergistic relationships exist among most regulating services, with “high-high” clustering areas located in northern, central-eastern, and southern mountains, forming ecological regulation core zones; while water purification services show significant trade-off relationships with most other services, revealing conflicts between ecological services under human activity background. SOM clustering identified five typical service bundles, whose dynamic evolution reflects the combined effects of natural system resilience and human activity reshaping. Geographic detector analysis results show that land use is the dominant factor in ES spatial differentiation, with the influence of socioeconomic driving forces showing a continuously strengthening trend. Based on the aforementioned results, this study proposes targeted ecological management strategies of “categorization and zoning,” guided by the identified ecosystem service bundles (ESBs). This provides a scientific basis for ensuring ecological security and territorial spatial management in the Lijiang River Basin.

## Research limitations and future directions

This study employed multiple spatial scales during data integration and analysis. While we strove to balance information retention with analytical feasibility, the fusion of data at different resolutions and the unified grid processing may still introduce uncertainties related to scale effects. Regarding the food production accounting, the method of converting all food products into grain yield equivalents before allocation reveals more information than the traditional approach based solely on grain yield. However, it does entail uncertainties associated with the spatial refinement in allocation. This study analyzed ESs from pixel and grid scales but did not assess them from administrative division unit perspectives; it mainly focused on ES supply capacity but did not systematically evaluate human activity demand characteristics for ESs, making it difficult to comprehensively reveal supply–demand–matching conditions. Additionally, this study focused on analyzing the spatiotemporal patterns, interaction relationships, and combinatorial characteristics of ESs, but did not conduct multi-scenario simulation predictions or further advance spatial planning practices such as ecological space identification and security pattern construction.

Future studies could employ higher-resolution datasets or appropriate methods to quantify scale effects, thereby further elucidating the impact of scale transformation on ecosystem service assessment and enhancing the precision of pattern identification and mechanistic interpretation. Additionally, we will further refine the raster allocation method for food production by attempting to incorporate precise data on feedlot distribution and farm locations, enabling more accurate spatial allocation of non-grain products. We will focus on constructing a dual assessment system based on pixels and administrative regions, establishing three typical development pathways: ecological protection priority, tourism development dominance, and natural evolution continuation, simulating ES supply–demand pattern evolution under different scenarios. Simultaneously, ecological source areas will be identified, and ecological resistance surfaces will be constructed, combining multiple dimensions including tourism activity intensity, construction land expansion, and topographic conditions, extracting ecological corridors and ecological restoration zones to provide a more operational scientific basis for ecological security pattern construction and precise territorial space governance in the Lijiang River Basin.

## Supplementary Information

Below is the link to the electronic supplementary material.

Supplementary Material 1

Supplementary Material 2

Supplementary Material 3

## Data Availability

All data sources used in this study, including their titles, repositories, persistent identifiers (DOIs/URLs), and corresponding references, are comprehensively listed in Table 1 of the main text.
