# Supplementary material for: Spatial heterogeneity, trade-offs, and bundle identification of ecosystem services in karst watersheds: a comprehensive assessment of the Lijiang River Basin
Source: Sci Rep. 2026 Apr 27;16:19425. doi: 10.1038/s41598-026-49739-x (PMC13287675; doi:10.1038/s41598-026-49739-x)
Supplement: Supplementary file 1 — Supplementary Material 1 [file 41598_2026_49739_MOESM1_ESM.docx]

Highlights:

- Evaluated ten ecosystem services in the Lijiang River karst basin using the InVEST model and spatial statistical methods.
- High-value ecosystem services aggregated to form distinct "ecological supply zones" and "pressure response zones".
- Revealed spatial patterns of trade-offs and synergies, analyzing the trade-off mechanism between water purification and other services.
- Identified five typical ecosystem service bundles via SOM clustering, uncovering ecosystem resilience and human-land relationship dynamics.
- Land use was the primary driver of spatial differentiation, with increasing influence from socio-economic factors.
