# Supplementary material for: Spatial heterogeneity, trade-offs, and bundle identification of ecosystem services in karst watersheds: a comprehensive assessment of the Lijiang River Basin
Source: Sci Rep. 2026 Apr 27;16:19425. doi: 10.1038/s41598-026-49739-x (PMC13287675; doi:10.1038/s41598-026-49739-x)
Supplement: Supplementary file 2 — Supplementary Material 2 [file 41598_2026_49739_MOESM2_ESM.docx]

**Supplementary Material**

**2.3.2 Spatiotemporal pattern evolution analysis of ESs**

This study carefully incorporates the natural, economic, and social realities of the Lijiang River Basin—such as the types and spatial distribution patterns of land use, and the economic profile of Guilin as an internationally renowned tourist destination where tourism serves as a pillar industry. These considerations form the basis for selecting assessment indicators and determining parameters in the ecosystem services evaluation process.

**(1) Biodiversity Conservation**

DEM, annual precipitation, mean annual temperature, and NPP data were normalized using the range method. The biodiversity maintenance service capacity index was then calculated in ArcGIS using the Raster Calculator according to the following formula ^1,2^:

$$\begin{aligned} S_{bio}={NPP}_{mean}\times F_{pre}\times F_{tem}\times\left( 1-F_{ait} \right)\#\left( 1 \right) \end{aligned}$$

Where: S_bio_ is the biodiversity maintenance service capacity index; NPP_mean_ is the mean annual net primary productivity; F_pre_ is the mean annual precipitation factor; F_tem_ is the mean annual temperature factor; F_alt_ is the altitude factor.

**(2) Habitat Quality**

The habitat quality module of the InVEST model was employed. Based on the land use data and road distribution data of the study area, the Threats Table^3^and Sensitivity Table (Table S_1. S_2) were established with reference to pertinent literature. Subsequently, the extent of habitats and vegetation types along with their degradation status were evaluated according to the following formula, resulting in the generation of a habitat quality map.

$$\begin{aligned} Q_{xj}=H_{j}\left( 1-\left( \frac{D_{xj}^{z}}{D_{xj}^{z}+k^{z}} \right) \right)\#\left( 2 \right) \end{aligned}$$

Where: Q_xj_ is the habitat quality for pixel x in land use type j, ranging from 0 to 1, with higher values indicating better quality. Hj is the habitat suitability for land use type j. K is the half-saturation constant.

Table S_1 Threats Table

| MAX_DIST | WEIGHT | THREAT | DECAY | DESCRIP |
| --- | --- | --- | --- | --- |
| 8000 | 0.7 | crops | linear | Cropland |
| 5000 | 0.6 | railway | linear | Railroad lines |
| 10000 | 1 | urban | exponential | Urban land |
| 5000 | 0.6 | village | exponential | Rural residential area |
| 2000 | 0.7 | roads | linear | Primary roads |
| 8000 | 0.8 | qtjsyd | exponential | Other construction land |

Table S_2 Sensitivity Table

| lucode | name | habitat | crops | railway | urban | village | roads | qtjsyd |
| --- | --- | --- | --- | --- | --- | --- | --- | --- |
| 11 | Paddy field | 0.6 | 0.3 | 0.4 | 0.7 | 0.6 | 0.4 | 0.4 |
| 12 | Dry farmland | 0.4 | 0.3 | 0.6 | 0.6 | 0.6 | 0.6 | 0.4 |
| 21 | Forestland | 1 | 0.8 | 3 | 0.85 | 0.9 | 0.65 | 0.6 |
| 22 | Shrubland | 1 | 0.4 | 4 | 0.6 | 0.65 | 0.6 | 0.5 |
| 23 | Sparse woodland | 1 | 0.9 | 5 | 0.8 | 0.9 | 0.5 | 0.6 |
| 24 | Other forest land | 1 | 0.9 | 0.7 | 0.85 | 0.85 | 0.7 | 0.6 |
| 31 | High-coverage grassland | 0.8 | 0.4 | 0.35 | 0.6 | 0.55 | 0.35 | 0.5 |
| 32 | Medium-coverage grassland | 0.7 | 0.5 | 0.4 | 0.7 | 0.5 | 0.4 | 0.6 |
| 33 | Low-coverage grassland | 0.6 | 0.5 | 0.3 | 0.6 | 0.5 | 0.3 | 0.6 |
| 41 | Rivers and canals | 0.8 | 0.7 | 0.6 | 0.6 | 0.5 | 0.6 | 0.8 |
| 42 | Lakes | 0.9 | 0.7 | 0.6 | 0.75 | 0.65 | 0.6 | 0.8 |
| 43 | Reservoirs and ponds | 0.7 | 0.7 | 0.6 | 0.6 | 0.5 | 0.6 | 0.8 |
| 45 | Tidal flat | 0.6 | 0.7 | 0.3 | 0.7 | 0.65 | 0.3 | 0.7 |
| 46 | Sandy beach | 0.6 | 0.7 | 0.3 | 0.7 | 0.5 | 0.3 | 0.7 |
| 51 | Urban land | 0 | 0 | 0 | 0 | 0 | 0 | 0 |
| 52 | Rural residential area | 0 | 0 | 0 | 0 | 0 | 0 | 0 |
| 53 | Other construction land | 0 | 0 | 0 | 0 | 0 | 0 | 0 |
| 64 | Marshland | 0.5 | 0.4 | 17 | 0.4 | 0.6 | 0.3 | 0.3 |
| 65 | Bare soil | 0 | 0 | 0 | 0 | 0 | 0 | 0 |
| 66 | Bare rock land | 0 | 0 | 0 | 0 | 0 | 0 | 0 |

**(3) Food Production**

Food production service, primarily supplied by cropland, forestland, and grassland, provides humans with agricultural products, cash crops, and livestock products. Given the positive correlation between food yield and NDVI ^4^, this study integrated data on agricultural, livestock, and aquatic products from statistical yearbooks. The production capacity of cropland, forestland, and grassland was spatially allocated using a weighting scheme based on the NDVI index. All products were uniformly converted into standard grain equivalent yield ^5^, enabling the spatial representation of supply service ^6^. The calculation formula is as follows^7,8^:

$$\begin{aligned} {FS}_{i}=\left\{ \begin{aligned} {FS}_{i}\times\frac{{NDVI}_{i}}{{NDVI}_{SUM}}\left( for farmland, forestland, grassland \right) \\ {FS}_{i}\times\frac{A_{o}}{A_{SUM}} \left( for water \right) \end{aligned} \right.\#\left( 3 \right) \end{aligned}$$

Where: G_i_ is the grain, meat, milk, or aquatic product in grid i. FS_sum_ is the total supply of each product in the study area. NDVI_i_ is the NDVI value in grid i. NDVI_sum_ is the sum of NDVI for cropland, forestland, and grassland in the study area. A_0_ is the area of one grid (1 km²).A_sum_ is the total water area in the study area.

In this context, it is essential to clarify that the "food production service" assessed in this study aims to evaluate the total supporting capacity of ecosystems for the human food supply system. This encompasses not only the direct output of grain and cash crops from croplands but also includes livestock products dependent on fodder supplied by forest and grassland ecosystems, as well as aquatic products from water bodies. Accordingly, during spatial allocation, livestock production was distributed to cropland, forestland, and grassland based on NDVI-weighted allocation, thereby representing the indirect contribution of these ecosystems to meat and dairy production through fodder provision and grazing space. Aquatic product yields were allocated to water bodies based on area. This approach allows for a more comprehensive reflection of the ecological foundation of the regional food supply system. However, it also implies that the results represent the "integrated supporting potential" of ecosystems for food production, rather than direct "crop yield."

**(4) Water Conservation**

Water conservation was estimated by modifying the water yield based on the surface runoff coefficient ^9^. The difference between water yield and surface runoff was used as the water conservation quantity ^10^, providing a more accurate reflection of the ecosystem's capacity to regulate water resources. The main calculation formulas are:

$$\begin{aligned} \boldsymbol{W}_{x}=\boldsymbol{Y}_{x}-\boldsymbol{R}_{x}\#\left( 4 \right) \end{aligned}$$

$$\begin{aligned} \boldsymbol{Y}_{x}=\boldsymbol{P}_{x}-{ET}_{x}\#\left( 5 \right) \end{aligned}$$

$$\begin{aligned} \boldsymbol{R}_{x}=\min\left( \boldsymbol{X}_{x}\times\boldsymbol{P}_{x}，\boldsymbol{Y}_{x} \right)\#\left( 6 \right) \end{aligned}$$

Where: Wx is the annual water conservation. Yx is the annual water yield. Rx is the annual surface runoff. Px is the annual precipitation. ETx is the annual evapotranspiration.

**(5) Soil Conservation**

Soil erosion was calculated using the Revised Universal Soil Loss Equation (RUSLE) model^11^ implemented via the sediment delivery ratio module of the InVEST model, based on data such as DEM and soil properties.

$$\begin{aligned} A_{c}=A_{p}-A_{r}=R\times K\times LS-R\times K\times LS\times C\times P\#\left( 7 \right) \end{aligned}$$

Where: A_c_ is the annual average soil retention per unit area. A_p_ and A_r_ are the potential and actual soil erosion amounts, respectively. R is the rainfall erosivity factor. K is the soil erodibility factor. LS is the topographic factor. P is the support practice factor. C is the cover-management factor. Values for C and P were adopted from the literature ^12^. The rainfall erosivity factor was calculated using the following formula ^13^:

$$\begin{aligned} R_{j}=0.0534\times P_{j}^{1.6548}\#\left( 8 \right) \end{aligned}$$

Where: P_j_ is the mean annual precipitation.

K represents the soil erodibility factor, which was calculated using the following formula^14,15^:

$$\begin{aligned} K=\left[ -0.01383+0.51575Kepic \right]\times0.1317\#\left( 9 \right) \end{aligned}$$

|  | $Kepic=\left\{ 0.2+0.3exp\left[ -0.0256sand\%\left( 1-\frac{silt\%}{100} \right) \right] \right\}\times\left( \frac{silt\%}{clay\%+silt\%} \right)^{0.3}\left[ 1-\frac{0.25\mathrm{OM}}{\mathrm{OM}+exp\left( 3.7-2.95\mathrm{OM} \right)} \right]\left\{ \left( 1-\frac{sand\%}{100} \right)+exp\left[ -5.51+22.9\left( 1-\frac{sand\%}{100} \right) \right] \right\}$ | (10) |
| --- | --- | --- |

Where: sand%, silt%, and clay% represent the proportion of sand, silt, and clay in the soil, respectively. OM represents the soil organic matter content. The data were sourced from the Soil data.

The soil erodibility factor was calculated in ArcGIS using the Raster Calculator, following the method described in the reference ^14^.

P and C represent the soil conservation practice factor and the vegetation cover factor, respectively. The values of C and P were determined based on the literature and adapted to the actual conditions of the study area^12^ (Table S_3). For other parameters, including the Threshold Flow Accumulation, Borselli K Parameter, Maximum SDR Value, Borselli ICO Parameter, and Maximum L Value, the system default values were retained.

Table S_3 Biophysical Table

| description | lucode | usle_c | usle_p |
| --- | --- | --- | --- |
| Paddy field | 11 | 0.1 | 0.01 |
| Dry farmland | 12 | 0.22 | 0.4 |
| Forestland | 21 | 0.006 | 1 |
| Shrubland | 22 | 0.04 | 1 |
| Sparse woodland | 23 | 0.01 | 1 |
| Other forest land | 24 | 0.04 | 0.7 |
| High-coverage grassland | 31 | 0.04 | 1 |
| Medium-coverage grassland | 32 | 0.04 | 1 |
| Low-coverage grassland | 33 | 0.04 | 1 |
| Rivers and canals | 41 | 0 | 0 |
| Lakes | 42 | 0 | 0 |
| Reservoirs and ponds | 43 | 0 | 0 |
| Tidal flat | 45 | 0 | 0 |
| Sandy beach | 46 | 0 | 0 |
| Urban land | 51 | 0 | 0 |
| Rural residential area | 52 | 0 | 0 |
| Other construction land | 53 | 0 | 0 |
| Marshland | 64 | 0 | 0 |
| Bare soil | 65 | 1 | 1 |
| Bare rock land | 66 | 0 | 0 |

**(6) Carbon Sequestration**

Based on land use data and carbon pool values for each land type in the Lijiang River Basin obtained from relevant literature ^16^, the total carbon storage was calculated using the carbon storage and sequestration module of the InVEST model. This module sums the aboveground, belowground, soil, and dead organic matter carbon pools for each land type. The Carbon Pools field values were defined according to relevant literature and the prevailing conditions within the study area ^16^, which are detailed in Table S_4.

Table S_4 Carbon Pools

| lucode | LULC_Name | C_above | C_below | C_soil | C_dead |
| --- | --- | --- | --- | --- | --- |
| 1 | Cropland | 13.5 | 2.7 | 96.59 | 1 |
| 2 | Forestland | 58.96555 | 19.97717 | 154.3441 | 3.224269 |
| 3 | Grassland | 5.01 | 13.53 | 117.06 | 1 |
| 4 | Water bodies | 0.21 | 0 | 0 | 0 |
| 5 | Construction land | 1.2 | 0.93 | 12.48 | 0 |
| 6 | Unused land | 19.52 | 3.9 | 0.86 | 0 |

Total carbon storage is calculated as follows ^17-19^:

$$\begin{aligned} C_{total}=C_{above}+C_{below}+C_{soil}+C_{dead}\#\left( 11 \right) \end{aligned}$$

Where: C_total_ denotes total carbon storage, i.e., the sum of all carbon pools in the ecosystem. C_above_ denotes aboveground biomass carbon, primarily the carbon in live plants above the surface (e.g., trunks, branches, leaves). C_below_ denotes belowground biomass carbon, primarily the carbon in plant roots. C_soil_ denotes the soil carbon pool, including carbon in soil organic matter and minerals. C_dead_ denotes litter/dead organic matter carbon, including surface litter and coarse woody debris.

**(7) Water Yield**

Water yield was estimated using the water yield module of the InVEST model, based on data including annual precipitation, annual actual evapotranspiration, bedrock depth, and land use ^20,21^. The method is grounded in the water balance principle, defining regional water yield as annual precipitation minus annual evapotranspiration ^22^. The calculation employs the following equation:

$$\begin{aligned} Y\left( x \right)=\left( 1-\frac{AET\left( x \right)}{P\left( x \right)} \right)\times P\left( x \right)\#\left( AUTONUM \backslash* Arabic 2 \right) \end{aligned}$$

Where: Y(x) is the annual water yield for pixel x. AET(x)is the annual actual evapotranspiration for that pixel. P(x) is the annual precipitation for that pixel.

The evapotranspiration component of the water balance is based on an expression of the Budyko curve^23,24^:

$$\begin{aligned} \frac{AET\left( x \right)}{P\left( x \right)}=1+\frac{PET\left( x \right)}{P\left( x \right)}-\left[ 1+\left( \frac{PET\left( x \right)}{P\left( x \right)} \right)^{\omega} \right]^{1/\omega}\#\left( 13 \right) \end{aligned}$$

Where: PET(x) is the potential evapotranspiration for pixel x. ω(x) is a non-physical parameter characterizing natural climate-soil properties.

Potential evapotranspiration PET(x) is defined as:

$$\begin{aligned} PET\left( x \right)=K_{c}\left( \mathcal{l}_{x} \right)\times ET_{0}\left( x \right)\#\left( 14 \right) \end{aligned}$$

Where: K_C_($\mathcal{l}$_X_)is the crop evapotranspiration coefficient for pixel x^25^. ET_0_(x)is the reference crop evapotranspiration coefficient for that pixel.

$$\begin{aligned} \omega\left( x \right)=Z\frac{AWC\left( x \right)}{P\left( x \right)}+1.25\#\left( 15 \right) \end{aligned}$$

AWC(x) is the plant-available water content for pixel x, representing the soil's capacity to supply water for vegetation growth ^26^. It is calculated as the product of the Plant-Available Water Capacity (PAWC) and the minimum value between the constraining layer depth and the vegetation rooting depth. PAWC is calculated using the following formula **^27^**(Equation 16). As noted in the User's Guide, soil depth can serve as a proxy for the root-restricting layer depth. Accordingly, this study employed the Depth-to-bedrock map of China at a spatial resolution of 100 meters ^28^ to represent the depth of the root-restricting layer. Z is an empirical constant termed the "seasonal factor". After repeated comparisons between modeling results and data from local water resources bulletins, it was calibrated to 1 for this study. The parameters for each field in the biophysical table were established according to the User's Guide and the actual conditions of the study area, and are presented in tabular form (Table S_5).

| $PAWC=54.509-0.132sand\%-0.003\left( sand\% \right)^{2}-0.055silt\%-0.006\left( silt\% \right)^{2}-0.738clay\%+0.007\left( clay\% \right)^{2}-2.688OM\%+0.501\left( OM\% \right)^{2}$ | (16) |
| --- | --- |

Where: sand%, silt%, and clay% represent the proportion of sand, silt, and clay in the soil, respectively. OM represents the soil organic matter content. The data were sourced from the Soil data.

Table S_5 Biophysical Table

| description | lucode | root_depth | Kc | LULC_veg |
| --- | --- | --- | --- | --- |
| Paddy field | 11 | 2100 | 0.7 | 1 |
| Dry farmland | 12 | 2000 | 0.65 | 1 |
| Forestland | 21 | 5200 | 1 | 1 |
| Shrubland | 22 | 5200 | 0.95 | 1 |
| Sparse woodland | 23 | 5200 | 0.93 | 1 |
| Other forest land | 24 | 5200 | 0.93 | 1 |
| High-coverage grassland | 31 | 2600 | 0.85 | 1 |
| Medium-coverage grassland | 32 | 2300 | 0.65 | 1 |
| Low-coverage grassland | 33 | 2000 | 0.65 | 1 |
| Rivers and canals | 41 | 100 | 1 | 0 |
| Lakes | 42 | 100 | 1 | 0 |
| Reservoirs and ponds | 43 | 100 | 1 | 0 |
| Tidal flat | 45 | 1000 | 1 | 0 |
| Sandy beach | 46 | 1000 | 1 | 0 |
| Urban land | 51 | 100 | 0.3 | 0 |
| Rural residential area | 52 | 100 | 0.2 | 0 |
| Other construction land | 53 | 100 | 0.3 | 0 |
| Marshland | 64 | 300 | 1 | 0 |
| Bare soil | 65 | 300 | 0.2 | 0 |
| Bare rock land | 66 | 300 | 0.2 | 0 |

**(8) Recreation Service**

The recreational services within the study area were simulated using the Recreation Opportunity Spectrum (ROS) model^29,30^. Recreational services represent non-material benefits that humans derive from nature, contributing to physical and psychological well-being^31^. Consequently, the provision of these services is influenced not only by the intrinsic attributes of ecosystems but also by factors such as transportation accessibility and geographical location. This study posits that in the Lijiang River Basin, where most scenic attractions are centered on the karst landscape of "Guilin's mountains and waters," the natural endowment is a primary factor attracting tourists and providing recreational value. Furthermore, in the topographically complex southwestern region of China, transportation accessibility is also a significant factor affecting service provision. Generally, higher levels of naturalness and accessibility, coupled with greater scenic spot and population density, correspond to a higher supply of recreational services. Therefore, an evaluation framework was constructed based on four dimensions: naturalness, accessibility, scenic spot density, and population density^32^.

First, within ArcGIS 10.8, land use data were manually reclassified and scored from 1 to 5, where 5 represents the highest degree of naturalness. Specifically, forests, water bodies, and wetlands were assigned a score of 5; grasslands and shrublands, 4; croplands and orchards, 3; bare land and sparse vegetation, 2; and urban, industrial, mining, and residential areas, 1.

Second, accessibility was scored based on the distance to transportation routes using ArcGIS 10.8: 0-500 meters was assigned 5 points; 500-1000 meters, 4 points; 1000-3000 meters, 3 points; 3000-5000 meters, 2 points; and distances greater than 5000 meters, 1 point.

Third, a kernel density analysis was performed on scenic spot data. Considering the characteristics of the study area, the search radius was set to 20,000 meters.

Fourth, population data were processed. The population density for the initial observation year was first classified into five categories using the natural breaks (Jenks) method. To ensure consistency, this classification scheme was then applied to the population density data of the subsequent four observation years for categorization and scoring. The category with the highest population density was assigned 5 points, and the category with the lowest density was assigned 1 point.

After processing these four datasets, the indicator weights were determined using a combined method of the entropy weight method and the analytic hierarchy process (AHP), with each method contributing 50% to the final weight. The spatial quantification of recreational service capacity was ultimately achieved through raster overlay.

**(9) Water Purification (Nitrogen, Phosphorus)**

This study employed the Nutrient Delivery Ratio (NDR) module of the InVEST model to assess the retention capacity for nitrogen and phosphorus, based on data including land use and DEM. The model simulates the transport of nutrients from source areas to water bodies by considering flow pathways derived from the DEM and the nutrient retention efficiency of different land use types. The purification service was quantified as the difference between the nutrient load and its final export.

During the analysis, parameters for the Biophysical Table were set with reference to the literature (Table S_6) ^33^, alongside watershed-level parameters such as the flow accumulation threshold and the Borselli k coefficient. Considering the local topography and the status of agricultural development in the study area, the Subsurface Critical Length (Nitrogen) and Subsurface Maximum Retention Efficiency (Nitrogen) were set to 300 and 0.7, respectively.

Table S_6 Biophysical Table

| description | lucode | load_p | eff_p | crit_len_p | load_n | eff_n | crit_len_n | Proportion  _subsurface_n |
| --- | --- | --- | --- | --- | --- | --- | --- | --- |
| Cropland | 1 | 5.75 | 0.25 | 150 | 24.2 | 0.25 | 150 | 0 |
| Forestland | 2 | 0.28 | 0.7 | 150 | 3.68 | 0.7 | 150 | 0 |
| Grassland | 3 | 0.55 | 0.4 | 150 | 8.5 | 0.4 | 150 | 0 |
| Water bodies | 4 | 0.01 | 0.05 | 150 | 0.01 | 0.05 | 150 | 0 |
| Construction land | 5 | 3.85 | 0.05 | 150 | 14.5 | 0.05 | 150 | 0 |
| Unused land | 6 | 0.01 | 0.25 | 150 | 0.01 | 0.25 | 150 | 0 |

**2.3.3** **ES correlation analysis**

**(1) Hot and Cold Spot Identification of ESs**

To reveal the spatial clustering patterns of each ecosystem service, this study employed spatial autocorrelation analysis. First, service values were aggregated based on 1-km² grid units. Global spatial autocorrelation was assessed using the Global Moran's I index. This statistic identifies whether services exhibit clustered, dispersed, or random distribution patterns at the regional scale by comparing the similarity of attribute values among neighboring spatial units. Its calculation formula is as follows:

$$\begin{aligned} I=\frac{N}{W}\frac{\sum_{i} \sum_{j} W_{ij}\left( x_{i}-\bar{x} \right)\left( x_{j}-\bar{x} \right)}{\sum_{i} \left( x_{i}-\bar{x} \right)^{2}}\#\left( 17 \right) \end{aligned}$$

Where: N is the total number of spatial units; xi and xj are the observed values at spatial units I and j; x̄ is the mean of all observed values; W_ij_ is an element of the spatial weight matrix quantifying the spatial relationship between units i and j; W is the sum of all spatial weights W_ij_. An I>0 indicates positive spatial autocorrelation (i.e., clustering of high or low values); I<0 indicates negative spatial autocorrelation (i.e., a checkerboard pattern of high and low values); I=0 suggests no significant spatial autocorrelation.

To further identify local spatial cluster types, hotspot analysis was conducted using the Getis-Ord Gi* statistic, calculated as:

$$\begin{aligned} G_{i}^{*}=\frac{\sum_{j} W_{ij}x_{j}-\bar{x}\sum_{j} W_{ij}}{S\sqrt{\frac{\left[ N\sum_{j} {W_{ij}}^{2}-\left( \sum_{j} W_{ij} \right)^{2} \right]}{N-1}}}\#\left( 18 \right) \end{aligned}$$

Where: $\bar{x}=\frac{\sum_{j} x_{i}}{N}$，$S=\sqrt{\frac{\sum_{j} x_{j}^{2}}{N}-\bar{x}^{2}}$

Statistically significant hotspots (clusters of high values) and coldspots (clusters of low values) were identified based on the z-score and corresponding p-value of each unit.

**(2) Trade-off and synergy analysis among ESs**

To clarify the interaction mechanisms among ecosystem services, Spearman's rank correlation coefficient was used in RStudio (R 4.5.1) to analyze pairwise relationships between services. This coefficient, which does not assume a specific data distribution and is robust to outliers ^34^, reflects the direction and strength of the relationship between service pairs ^35,36^ and is thus widely used in ES trade-off and synergy studies ^37-41^. A positive result indicates a synergistic relationship (mutual enhancement), while a negative result indicates a trade-off relationship (one increases at the expense of the other) ^36^. A coefficient near zero suggests the two services are independent.

Based on Cohen's guidelines^42^ and their widespread application in environmental research^43,44^, the absolute value of the correlation coefficient was interpreted as follows to indicate strength: negligible (0.00–0.19), weak (0.20–0.39), moderate (0.40–0.59), strong (0.60–0.79), and very strong (0.80–1.00).

To further reveal the spatial heterogeneity of trade-off/synergy relationships, bivariate local spatial autocorrelation analysis in Geoda software was employed to identify local cluster types for each ES pair. 'High-High' and 'Low-Low' clusters represent spatial synergy, whereas 'High-Low' and 'Low-High' clusters reflect spatial trade-offs. This analysis, conducted on the Geoda platform, effectively revealed the local patterns and spatial dependence of interactions between services.

**2.3.4 ES bundle identification**

To reveal the synergistic/trade-off relationships among ecosystem services (ESs) and their spatial aggregation patterns within the Lijiang River Basin, an unsupervised machine learning algorithm—Self-Organizing Maps (SOM)—was employed to identify and analyze the spatiotemporal dynamics of Ecosystem Service Bundles (ESBs). The entire analytical workflow was completed on the RStudio (R 4.5.1) platform, with specific steps as follows:

(1) Data Preprocessing and Baseline Year Selection

The analysis dataset consisted of the biophysical quantity data for ten ESs calculated at a 1km×1km grid scale across five observation years. To ensure comparability of multi-temporal results and avoid classification bias caused by inter-annual variations in data distribution, a "fixed classification framework" strategy was adopted. The year 2010 was selected as the baseline training year, primarily based on the following considerations: (1) This year is situated in the middle of the study time series, thus providing a representative snapshot of the overall state of ESs during the period; (2) The data quality for 2010 was excellent, with no missing values, making it a suitable reference benchmark.

(2) SOM Model Training and Parameter Settings

The kohonen R package was used for the SOM analysis.

The ES data for 2010 were standardized using Z-score normalization.

A 2D rectangular topological grid was created with dimensions set to xdim = 6 and ydim = 1, resulting in a total of 6 neurons.

The random seed was fixed using set.seed(2022) to ensure the complete reproducibility of the analysis.

Training control parameters: When calling the core function som(), the data (X), grid (g), and initial neighborhood radius (radius = 1) were specified, while other training parameters were kept at their default values.

(3) Determination and Validation of the Number of Service Bundles

The final number of ESBs was determined through a combination of data-driven and statistical validation methods:

Preliminary Visual Interpretation: Upon completion of training, the U-Matrix and sample distance plots of the SOM were generated to preliminarily observe the self-organized structure of the data within the neural network and identify potential cluster boundaries.

Quantitative Optimization via Silhouette Index: The weight vectors of the 6 neurons from the SOM model were extracted and subjected to hierarchical clustering. To objectively determine the optimal number of clusters, the Silhouette Index was calculated for cluster numbers (k) ranging from 2 to 6. The results indicated that the Silhouette Index peaked when k = 5, suggesting that partitioning the data into 5 ESBs was statistically the most reasonable.

Interpretation of Ecological Characteristics: Based on the k=5 partition scheme, radar charts were plotted. The five identified ESBs exhibited clear, significantly distinct, and ecologically meaningful patterns in their functional composition, confirming the practical interpretability of this classification outcome.

In summary, this study determined the number of ESB types in the Lijiang River Basin to be five.

(4)Multi-temporal Data Mapping and Spatiotemporal Dynamic Analysis

To analyze the spatiotemporal evolution of ESBs, the ES data for the years 2000, 2005, 2015, and 2020 were standardized using the mean and standard deviation from the 2010 baseline period. This step aimed to eliminate dimensional differences and ensure consistency with the training benchmark. Subsequently, the kohonen::map() function was used to project the standardized data from each year onto the pre-trained 2010 SOM model. Based on the established classification rules, the spatial distribution of ESBs under a unified classification standard was obtained for all years. This method fundamentally guaranteed the comparability of classification results across different time periods, allowing the subsequent analysis of area changes and spatial pattern evolution to genuinely reflect dynamic ecological processes.

Finally, a comprehensive analysis utilizing radar charts, spatial distribution maps of ESBs, and Sankey diagrams (created using Origin 2025) systematically revealed the functional characteristics, spatial patterns, and dynamic transition pathways of each ESB from 2000 to 2020.


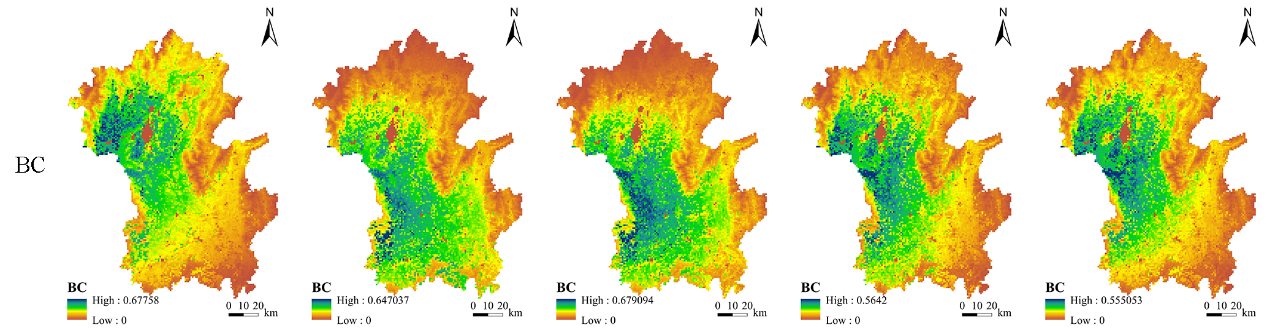


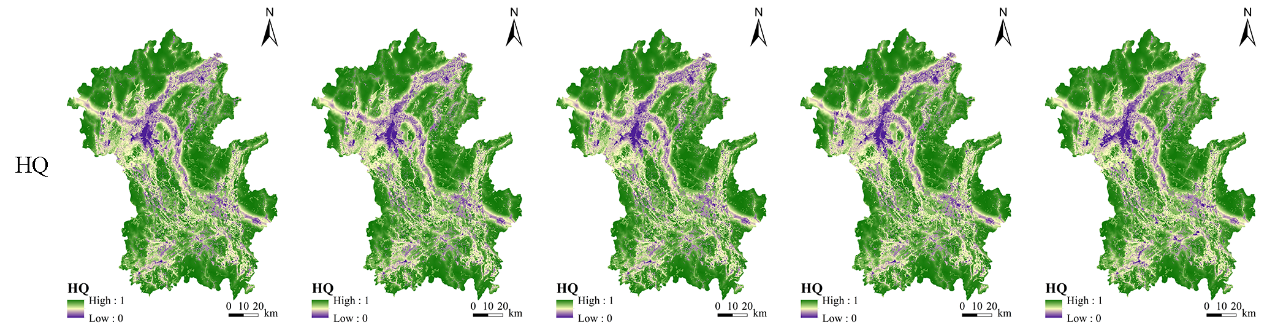


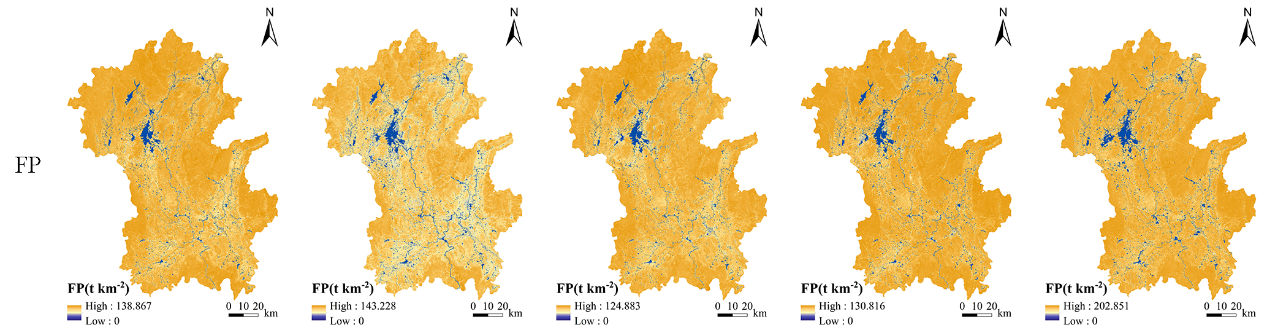


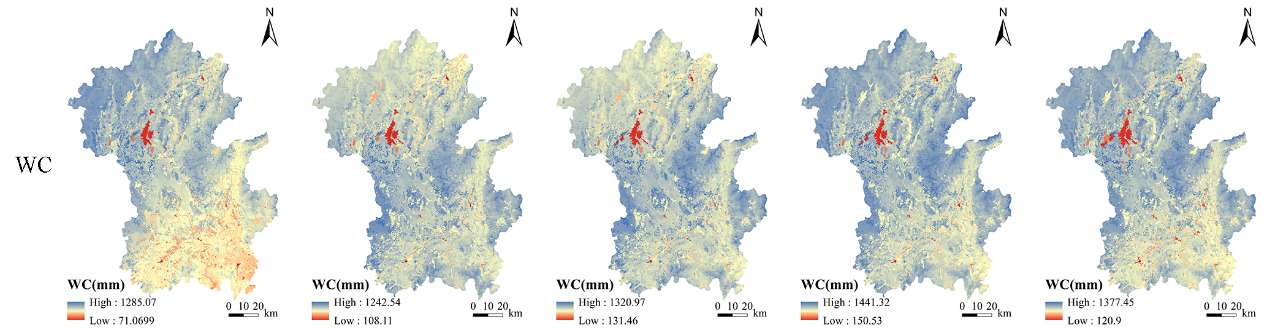


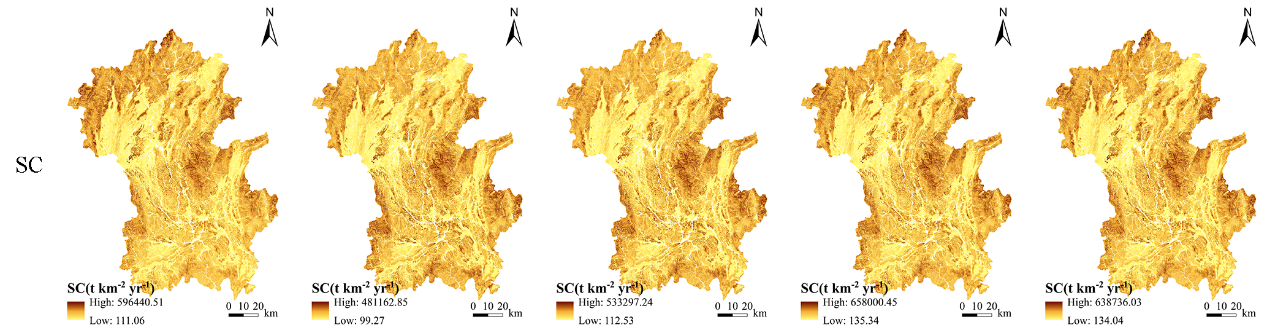


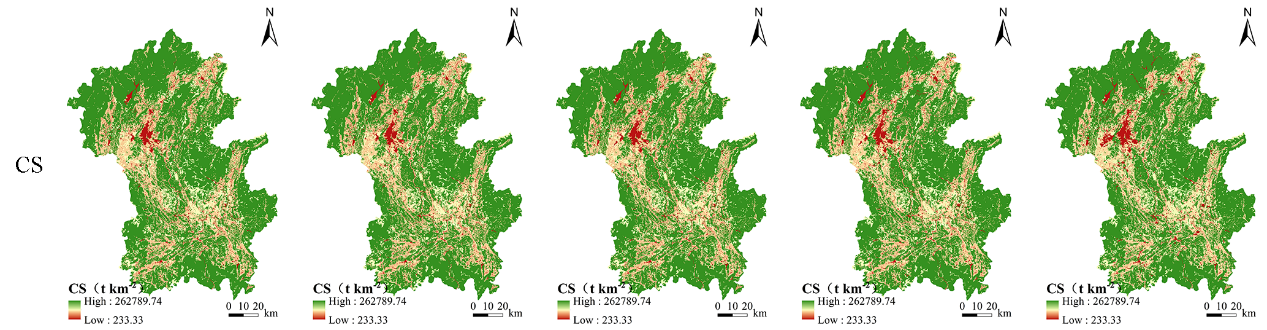


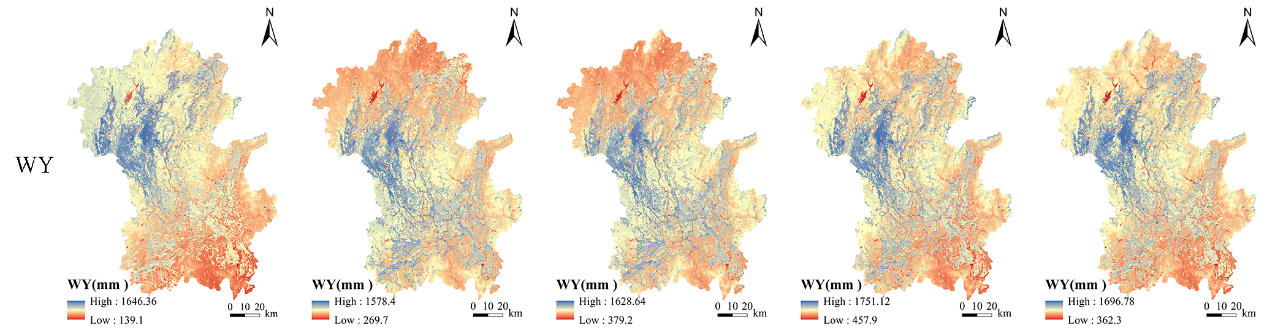


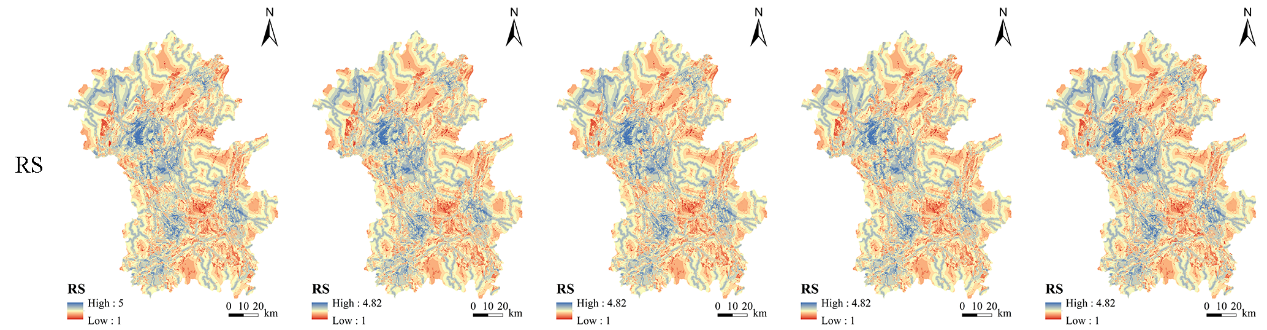


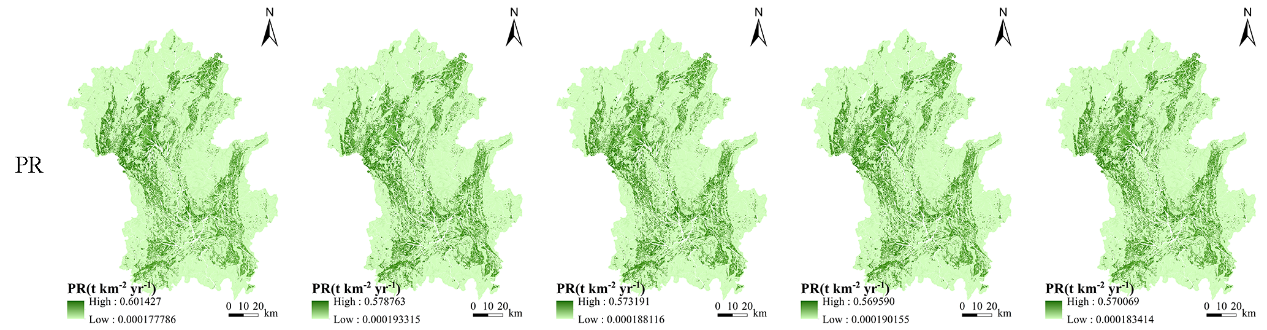


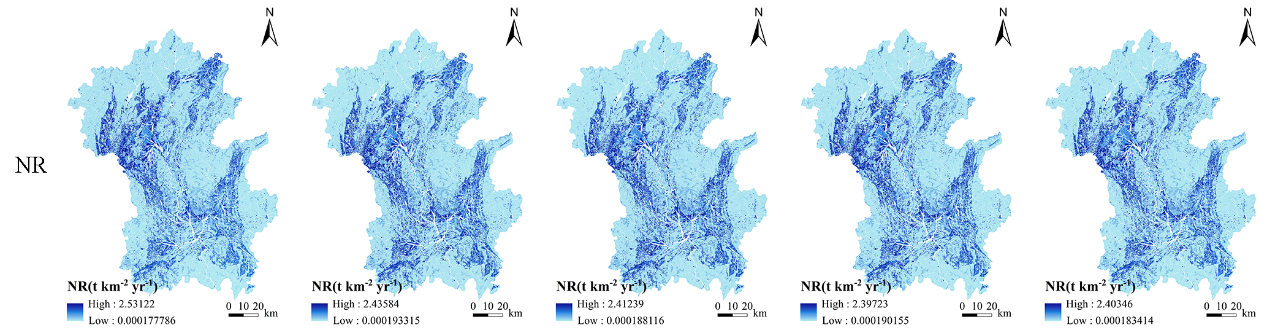


Fig.S1 Spatiotemporal distribution of each ecosystem service

**Note:** The panels are arranged in chronological order (2000, 2005, 2010, 2015, 2020) from left to right.

In this figure, the subplots for HQ, SC, CS, WY, PR and NR were generated using InVEST 3.16.1 (<https://naturalcapitalproject.stanford.edu/software/invest>) following the methods described in the “Research Methods” section, and were then processed using ArcGIS 10.8 (<http://www.esri.com/software/arcgis>). The subplots for other service types were generated using ArcGIS 10.8 (<http://www.esri.com/software/arcgis>) as described in the “Research Methods” section. All subplots were then composited into this figure using Adobe Photoshop 2020 ([https://www.adobe.com/cn](https://www.adobe.com/cn)). This figure was created independently by the authors.

BC, Biodiversity Conservation; HQ, Habitat Quality; FP, Food Production; WC, Water Conservation; SC, Soil Conservation; CS, Carbon Sequestration; WY, Water Yield; RS, Recreational Services; NR, Nitrogen Retention; PR, Phosphorus Retention


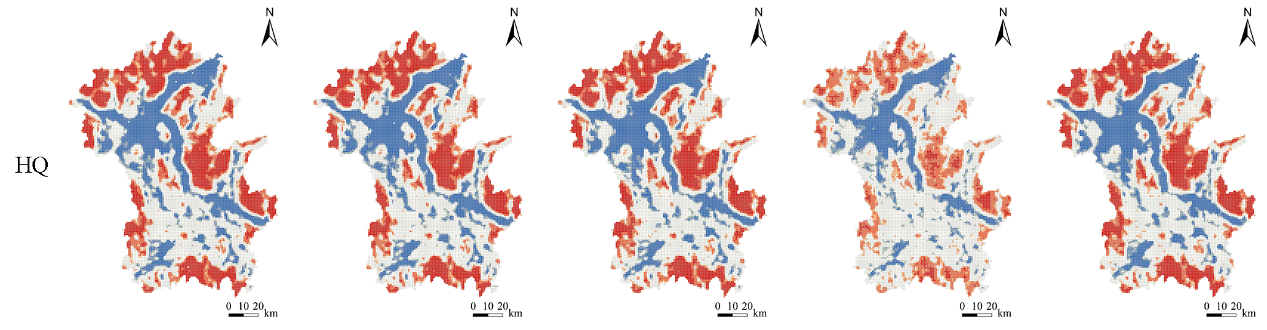


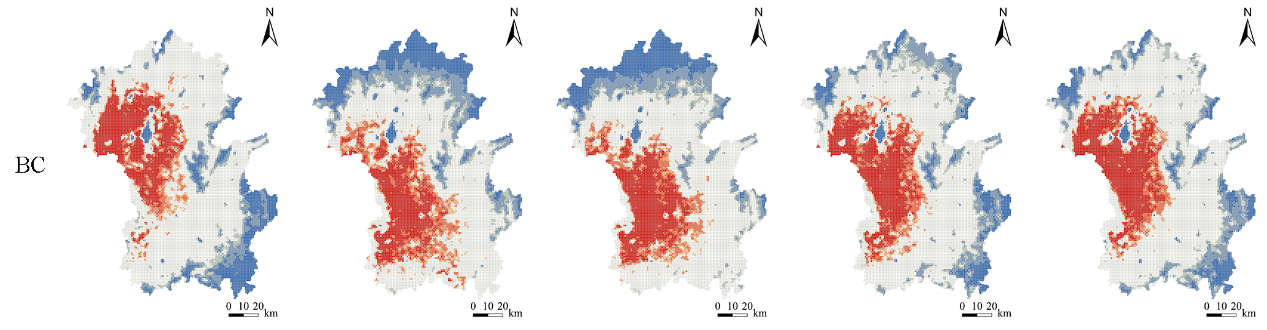


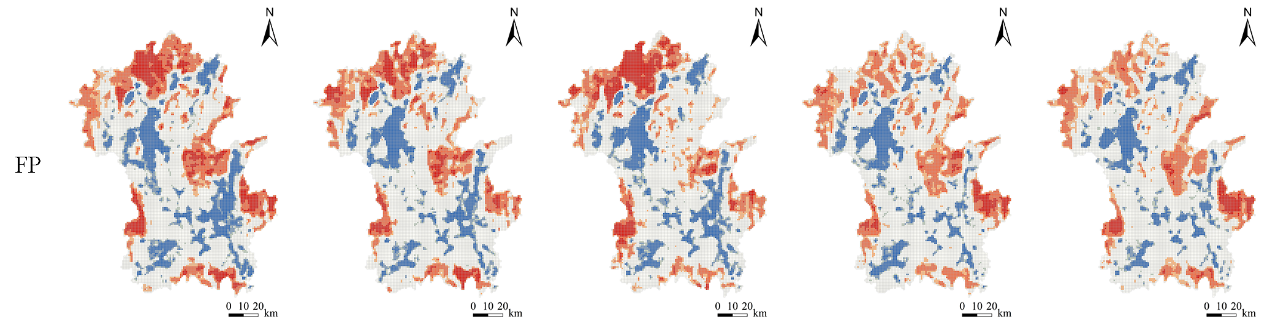


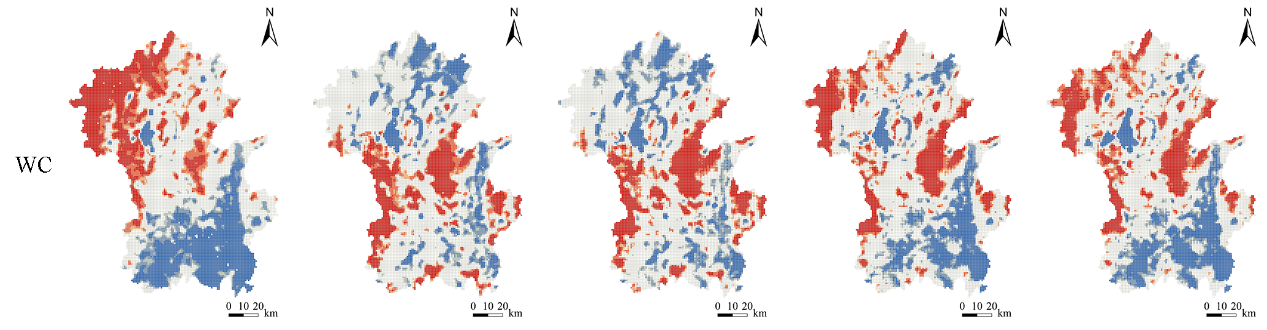


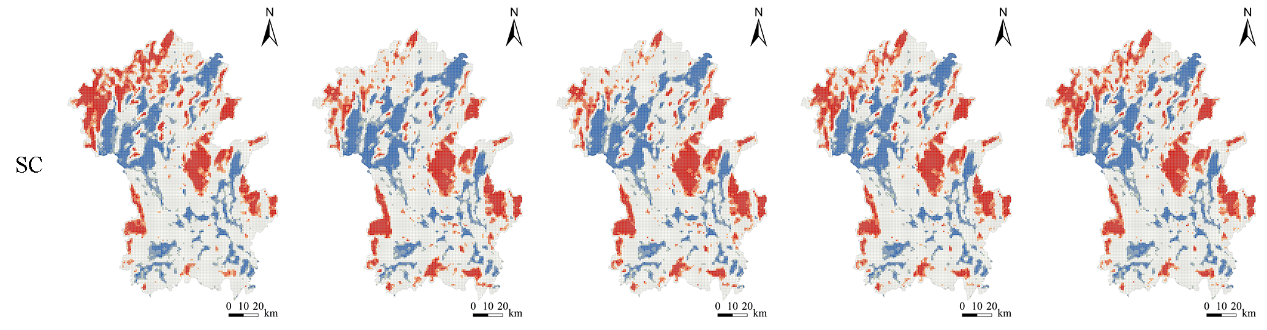


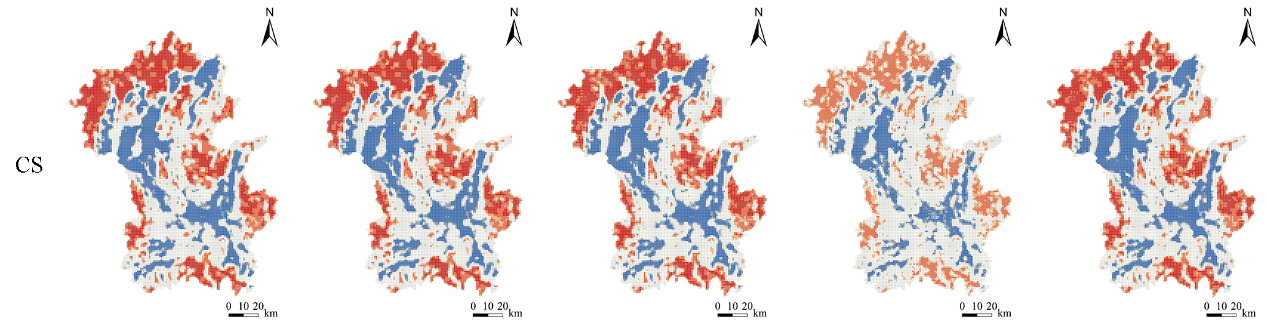


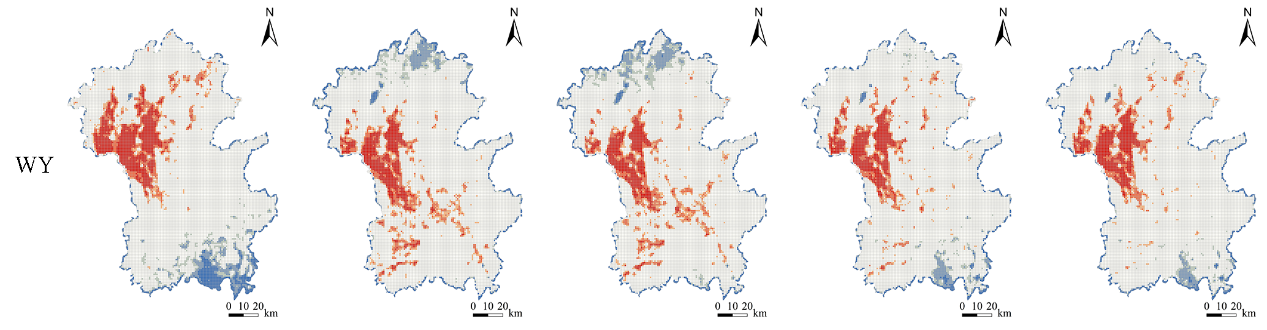


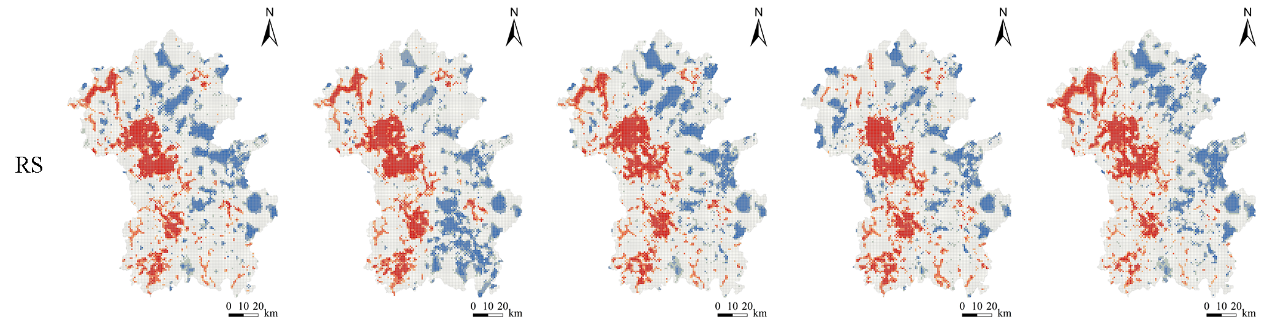


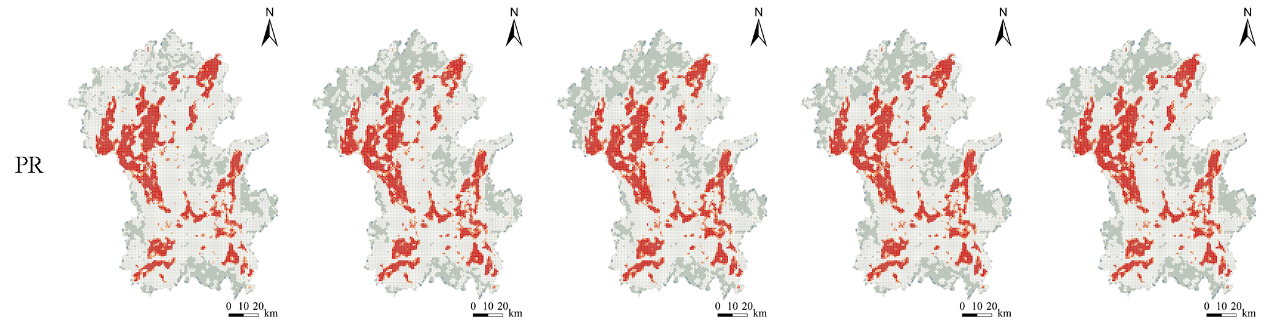


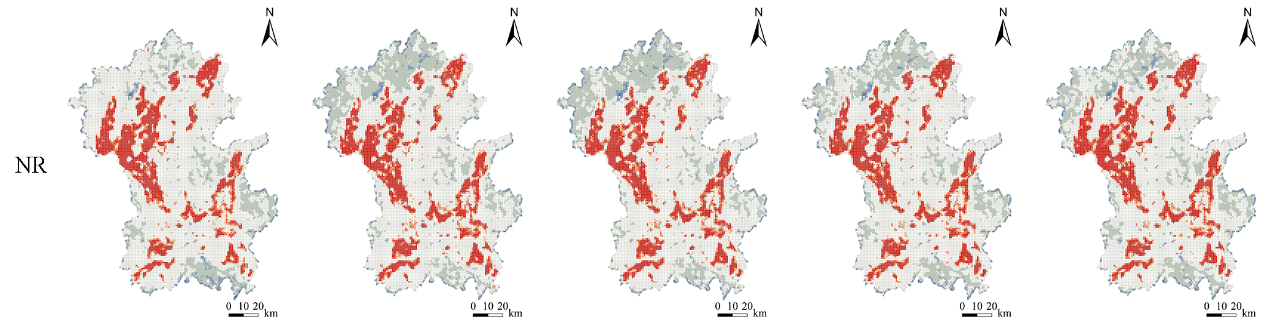


Fig. S2 Hot and cold spot distribution of each ecosystem service

**Note:** The panels are arranged in chronological order (2000, 2005, 2010, 2015, 2020) from left to right.

The subplots in this figure were generated using ArcGIS 10.8 (<http://www.esri.com/software/arcgis>), and all subplots were then composited into this figure using Adobe Photoshop 2020 (<https://www.adobe.com/cn>). This figure was created independently by the authors.

BC, Biodiversity Conservation; HQ, Habitat Quality; FP, Food Production; WC, Water Conservation; SC, Soil Conservation; CS, Carbon Sequestration; WY, Water Yield; RS, Recreational Services; NR, Nitrogen Retention; PR, Phosphorus Retention


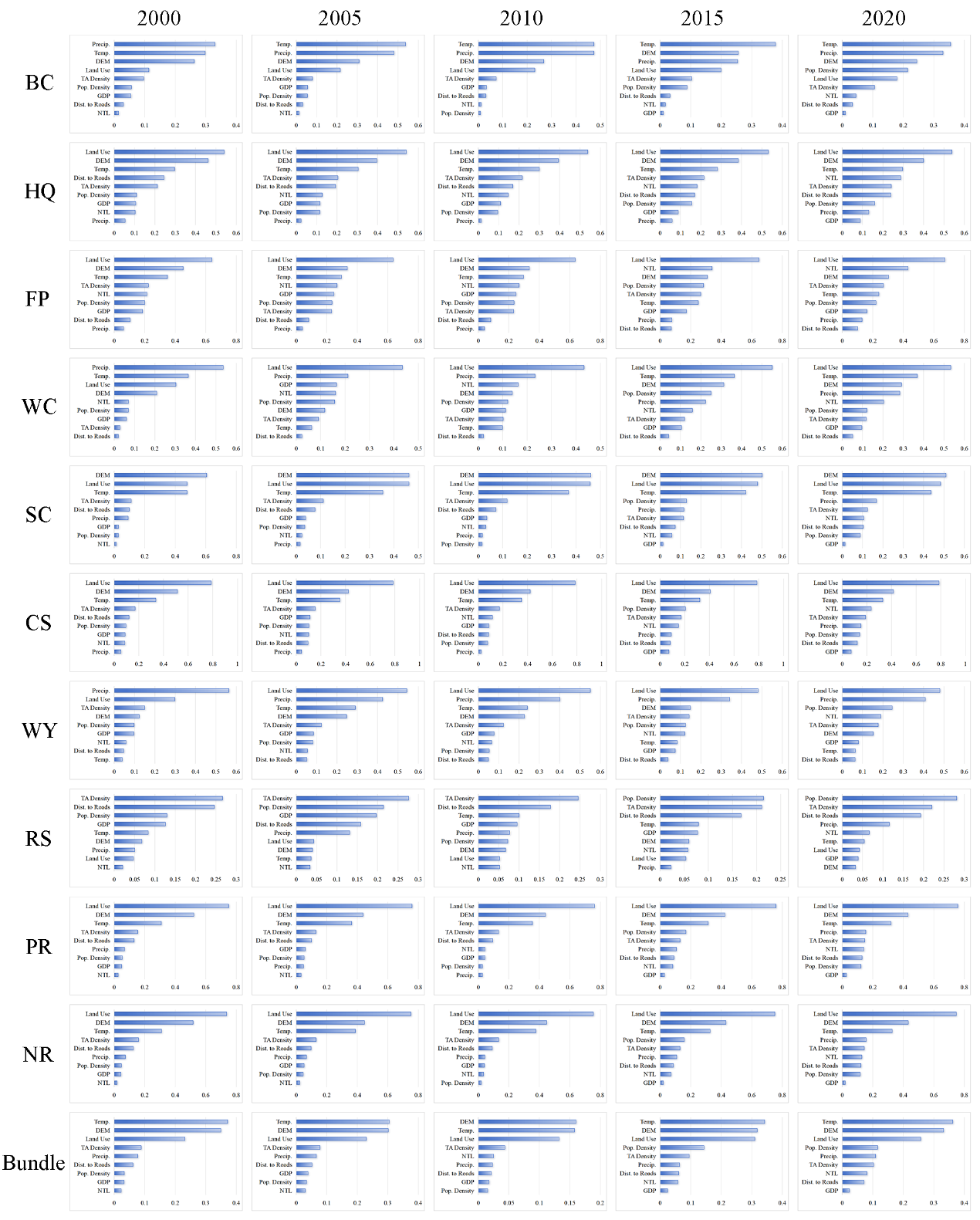
Fig.S3. Driving factors of each ecosystem service and ecosystem service bundles

**Note:** The subplots in this figure were generated using Excel 2019 (<https://www.office.com/>), and all subplots were then composited into this figure using Adobe Photoshop 2020 (<https://www.adobe.com/cn>). This figure was created independently by the authors.

**References**

1. Sun, L. H., Liu, H., Wang, D., Hao, H. G. Research on Ecological Security Pattern Construction Based on the Evaluationof Ecosystem Services and Eco-Environmental Sensitivity. *Research of Environmental Sciences.* **35**, 2508-2517, <https://doi:10.13198/j.issn.1001-6929.2022.10.11> (2022).

2. Tu, C., Luo, W. Q., Chen, Y. Q., Wu, Z. Y., Hu, Z. X., Liu, S. H., Ma, Q., Qin, L. T. Zoning management of karst landscape resources in Guilin based on ecosystem sensitivity and service function. *Geology in China.* **51**, 1839-1854 (2024).

3. Huang, G. J. Evaluation of Ecosystem Services in Karst Basin Based onInVEST Model, Guizhou Normal University, (2020).

4. Kuri, F. M., A. Murwira, K.S. Masocha, M. Predicting maize yield in Zimbabwe using dry dekads derived from remotely sensed vegetation condition index. *International Journal of Applied Earth Observation and Geoinformation.* **33**, 39-46, <https://doi:10.1016/j.jag.2014.04.021> (2014).

5. Yan, W. P. Spatial and Temporal Characteristics of Ecosystem Service Supply and Demand in the Upper Reaches of the Min River and Research on Ecologica Compensation, Southwest University Of Science And Technology, (2024).

6. Qiao, X. S., Y. Guo, J. Yang, Y. et al. Impacts of urban expansion on ecosystem services in different size cities of Zhengzhou metropolitan area. *Geographical Research.* **41**, 1913-1931 (2022).

7. Li, Q. et al. Spatial heterogeneity of ecosystem service bundles and the driving factors in the Beijing-Tianjin-Hebei region. *J. Clean. Prod.* **479**, <https://doi:10.1016/j.jclepro.2024.144006> (2024).

8. Fan, S. X., Yan, M., Yu, L. F., Chen, B. W. & Zhang, L. Integrating ecosystem service supply-demand and ecological risk assessment for urban planning: A case study in Beijing, China. *Ecol. Indicat.* **161**, <https://doi:10.1016/j.ecolind.2024.111950> (2024).

9. Shen, M. S., Liu, Y. Y., Zheng, H., Chen, J. Evaluation of Water Source Conservation Service Value and Its Spatial Transfer in Yangtze River Basin. *Journal of Changjiang River Scientific Research Institute.* **41**, 14-22+36 (2024).

10. Zhang, C. S., Fan, N., Liu, C. L., Xie, G. D. Spatio-temporal pattern and evolution of ecosystem water conservation in China from 1990 to 2018. *Acta Ecol Sin.* **43**, 5536-5545 (2023).

11. Jin, F. Y., W. Fu, J. Li, Z. Effects of vegetation and climate on the changes of soil erosion in the Loess Plateau of China. *Science of The Total Environment.* **773**, 145514, <https://doi:10.1016/j.scitotenv.2021.145514> (2021).

12. Xu, Y. Q., Shao, X. M. Estimation of soil erosion supported by GIS and RUSLE: A case study of Maotiaohe Watershed, Guizhou Province. *Journal of Beijing Forestry University.* 67-71, <https://doi:Cnki:Sun:Bjly.0.2006-04-013> (2006).

13. Zhang, W. B., Fu, J. S. Rainfall erosivity estimation under different rainfall amount. *Resources Science.* 35-41, <https://doi:Cnki:Sun:Zrzy.0.2003-01-005> (2003).

14. Zhou, P. Investigation on the Optimization of Territorial Space Pattern and Function Enhancement Path in Taihang Mountain Region, University of Chinese Academy of Sciences, (2020).

15. Zhao, X. Y., Wang, J. F., Li, Q. & Li, W. J. The function of soil conservation in Beisan River Basin based on InVEST model. *Journal of Shihezi University( Natural Science).* **40**, 487-496, <https://doi:10.13880/j.cnki.65-1174/n.2022.23.004> (2022).

16. Zhou, X. R., Wang, J. Y., Tang, L., He, W. & Li, H. Impact of Land Use Change on Carbon Storage Dynamics in the Lijiang River Basin, China: A Complex Network Model Approach. *Land.* **14**, <https://doi:10.3390/land14051042> (2025).

17. Peng, J. et al. Simulating the impact of Grain-for-Green Programme on ecosystem services trade-offs in Northwestern Yunnan, China. *Ecosyst. Serv.* **39**, <https://doi:10.1016/j.ecoser.2019.100998> (2019).

18. An, Q. M. et al. Spatio-temporal interaction and constraint effects between ecosystem services and human activity intensity in Shaanxi Province,China. *Ecol. Indicat.* **160**, <https://doi:10.1016/j.ecolind.2024.111937> (2024).

19. Xie, B. G., You, S. M. & Zhou, K. C. Spatiotemporal evolution and multiscenario simulation of cultivated land ecosystem services in the Dongting Lake Plain. *Sci. Rep.* **15**, <https://doi:10.1038/s41598-025-95960-5> (2025).

20. Dennedy-Frank, P. J., Muenich, R. L., Chaubey, I. & Ziv, G. Comparing two tools for ecosystem service assessments regarding water resources decisions. *J. Environ. Manag.* **177**, 331-340, <https://doi:10.1016/j.jenvman.2016.03.012> (2016).

21. He, L. J. et al. Exploring the interrelations and driving factors among typical ecosystem services in the Yangtze river economic Belt, China. *J. Environ. Manag.* **351**, <https://doi:10.1016/j.jenvman.2023.119794> (2024).

22. Fu, B. P. On the calculation of the evaporation from land surface (in Chinese). *Sci. Atmos. Sin.* **5**, 23– 31 (1981).

23. Fu, B. P. On the calculation of the evaporation from land surface. *Scientia Atmospherica Sinica.* **5**, 23-31 (1981).

24. Zhang, L. a. H., K. and Dawes, W. R. and Chiew, F. H. S. and Western, A. W. and Briggs, P. R. A rational function approach for estimating mean annual evapotranspiration. *Water Resources Research.* **40** (2004).

25. Allen, R. G. P., L.S. Raes, D. Smith, M. (1998).

26. Donohue, R. J. R., M. L. McVicar, T. R. Roots, storms and soil pores: Incorporating key ecohydrological processes into Budyko's hydrological model. *Journal of Hydrology.* **436-437**, 35-50 (2012).

27. Zhou, W. Z., Liu, G. H., Pan, J. J. Distribution of available soil water capacity in China. *Journal of Geographical Sciences.* 5-14, <https://doi:Cnki:Sun:Zgde.0.2005-01-000> (2005).

28. Yan, F. P., Wei, S. G., Zhang, J. & Hu, B. F. Depth-to-bedrock map of China at a spatial resolution of 100 meters. *Sci. Data.* **7**, <https://doi:10.1038/s41597-019-0345-6> (2020).

29. Boyd, S. W. B., R.W. Vol. 17 (ed Tourism Management) 557-566 (1996).

30. Huang, F. X. et al. Exploring the driving factors of trade-offs and synergies among ecological functional zones based on ecosystem service bundles. *Ecol. Indicat.* **146**, <https://doi:10.1016/j.ecolind.2022.109827> (2023).

31. Willis, C. Vol. 10 38-43 (Journal of Outdoor Recreation and Tourism-Research Planning and Management, 2015).

32. Maes, J. et al. Mainstreaming ecosystem services into EU policy. *Current Opinion in Environmental Sustainability.* **5**, 128-134, <https://doi:10.1016/j.cosust.2013.01.002> (2013).

33. Li, W., Zhao, Z. L., Lyu, S. S., Zhao, W. Q. Attenuation of Pollutants in Beipanjiang River Basin Calculated Using the InVEST Model. *Journal of Irrigation and Drainage.* **41**, 105-113, <https://doi:10.13522/j.cnki.ggps.2021471> (2022).

34. Zar, J. H. in Encyclopedia of Biostatistics (Wiley, 2005).

35. Sylla, M., Hagemann, N. & Szewranski, S. Mapping trade-offs and synergies among peri-urban ecosystem services to address spatial policy. *Environmental Science & Policy.* **112**, 79-90, <https://doi:10.1016/j.envsci.2020.06.002> (2020).

36. Wu, L. L., Sun, C. G. & Fan, F. L. Multi-criteria framework for identifying the trade-offs and synergies relationship of ecosystem services based on ecosystem services bundles. *Ecol. Indicat.* **144**, <https://doi:10.1016/j.ecolind.2022.109453> (2022).

37. Li, Y. & Luo, H. F. Trade-off/synergistic changes in ecosystem services and geographical detection of its driving factors in typical karst areas in southern China. *Ecol. Indicat.* **154**, <https://doi:10.1016/j.ecolind.2023.110811> (2023).

38. Wang, L. N., Yu, E. Y., Li, S., Fu, X. & Wu, G. Analysis of Ecosystem Service Trade-Offs and Synergies in Ulansuhai Basin. *Sustainability.* **13**, <https://doi:10.3390/su13179839> (2021).

39. Zeng, J. W. et al. Evaluating Trade-Off and Synergies of Ecosystem Services Values of a Representative Resources-Based Urban Ecosystem: A Coupled Modeling Framework Applied to Panzhihua City, China. *Remote Sensing.* **14**, <https://doi:10.3390/rs14205282> (2022).

40. Zhang, J. X. et al. Trade-offs and synergies of ecosystem services and their threshold effects in the largest tableland of the Loess Plateau. *Global Ecol. Conserv.* **48**, <https://doi:10.1016/j.gecco.2023.e02706> (2023).

41. Simeon, M. & Wana, D. Synergies and trade-offs among key ecosystem services in Maze National Park and its environs, southwestern Ethiopia. *Global Ecol. Conserv.* **57**, <https://doi:10.1016/j.gecco.2024.e03398> (2025).

42. Cohen, J. Statistical power analysis for the behavioral sciences. 2nd edn, (Lawrence Erlbaum Associates, 1988).

43. Bennett, E. M. P., G. D. Gordon, L. J. Understanding relationships among multiple ecosystem services. *Ecol. Lett.* **12**, 1394-1404 (2009).

44. Qiu, J. T., M. G. Spatial interactions among ecosystem services in an urbanizing agricultural watershed. *Proc. Natl Acad. Sci. USA.* **110**, 12149-12154 (2013).
