# Supplementary material for: Spatial heterogeneity, trade-offs, and bundle identification of ecosystem services in karst watersheds: a comprehensive assessment of the Lijiang River Basin
Source: Sci Rep. 2026 Apr 27;16:19425. doi: 10.1038/s41598-026-49739-x (PMC13287675; doi:10.1038/s41598-026-49739-x)
Supplement: Supplementary file 3 — Supplementary Material 3 [file 41598_2026_49739_MOESM3_ESM.docx]

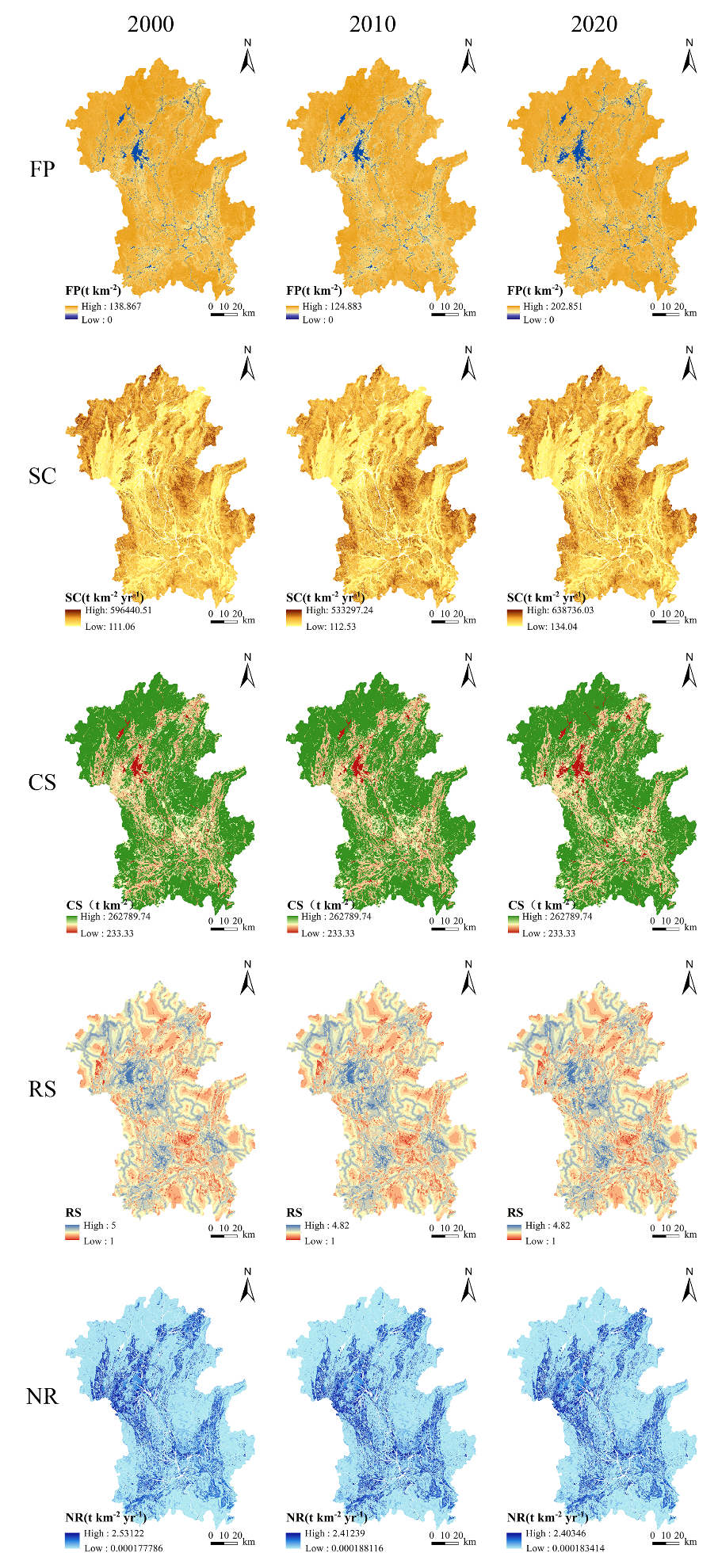


Fig 1. Spatiotemporal distribution of each ecosystem service( 3 observation years, 5 ecosystem services) (This integrated image corresponds to Figure 3 in the main text.)

In this integrated image, the subplots for SC, CS, and NR were generated using InVEST 3.16.1 (<https://naturalcapitalproject.stanford.edu/software/invest>) following the methods described in the “Research Methods” section, and were then processed using ArcGIS 10.8 (<http://www.esri.com/software/arcgis>). The subplots for other service types were generated using ArcGIS 10.8 (<http://www.esri.com/software/arcgis>) as described in the “Research Methods” section. All subplots were then composited into this integrated image using Adobe Photoshop 2020 ([https://www.adobe.com/cn](https://www.adobe.com/cn)). This figure was created independently by the authors.


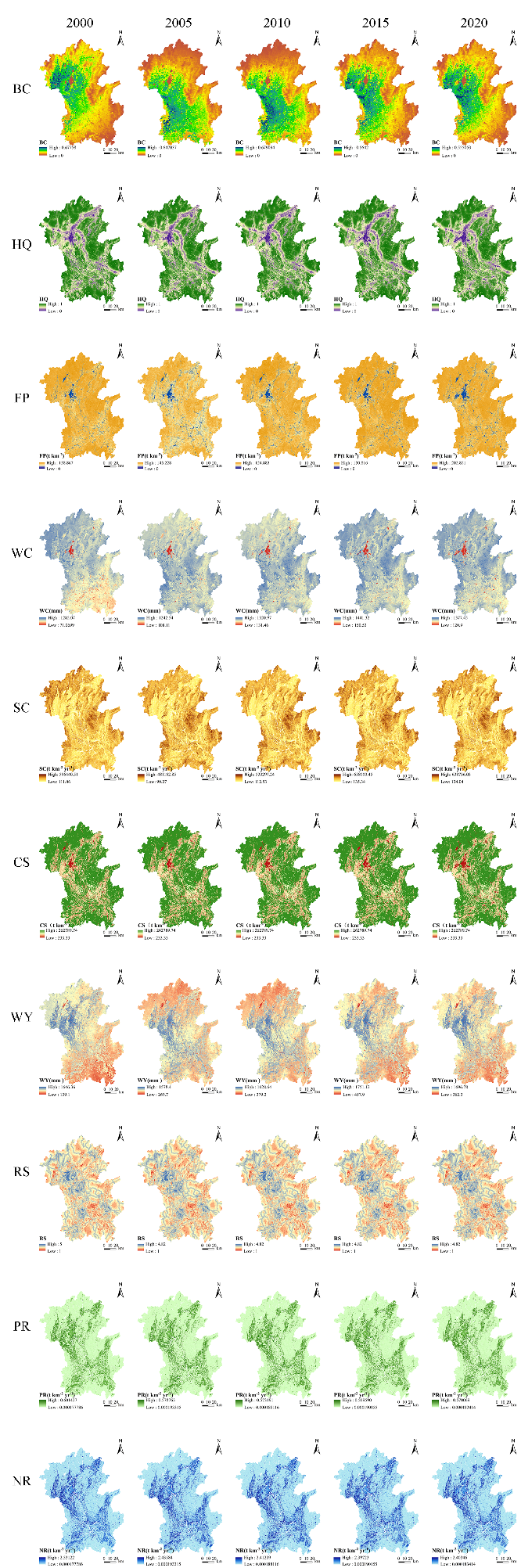


Fig 2. Spatiotemporal distribution of each ecosystem service( 5 observation years, 10 ecosystem services) (This integrated image corresponds to Figure S1 in the Supplementary Material.)

In this integrated image, the subplots for HQ, SC, CS, WY, PR and NR were generated using InVEST 3.16.1 (<https://naturalcapitalproject.stanford.edu/software/invest>) following the methods described in the “Research Methods” section, and were then processed using ArcGIS 10.8 (<http://www.esri.com/software/arcgis>). The subplots for other service types were generated using ArcGIS 10.8 (<http://www.esri.com/software/arcgis>) as described in the “Research Methods” section. All subplots were then composited into this integrated image using Adobe Photoshop 2020 (<https://www.adobe.com/cn>). This figure was created independently by the authors.


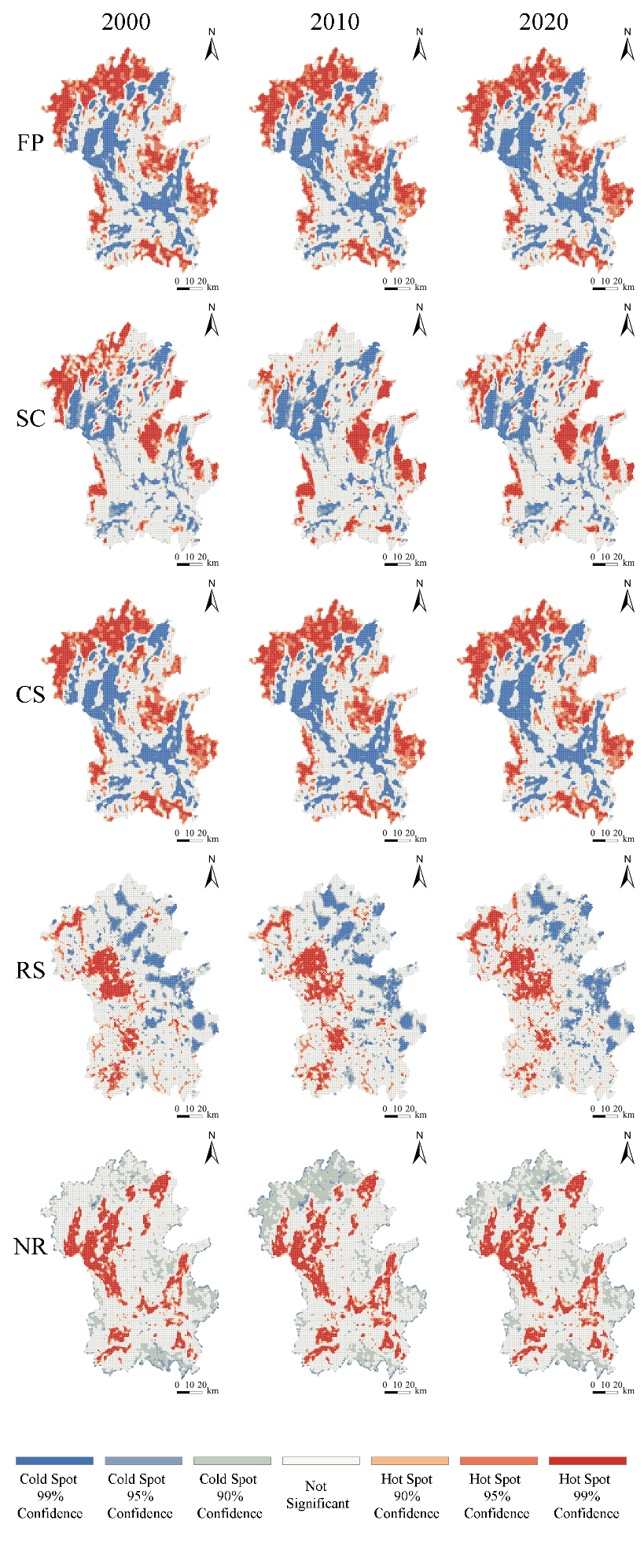


Fig 3. Hot and cold spot distribution of each ecosystem service( 3 observation years, 5 ecosystem services) (This integrated image corresponds to Figure 4 in the main text.)

The subplots in this integrated image were generated using ArcGIS 10.8 (<http://www.esri.com/software/arcgis>), and all subplots were then composited into this integrated image using Adobe Photoshop 2020 (<https://www.adobe.com/cn>). This figure was created independently by the authors.


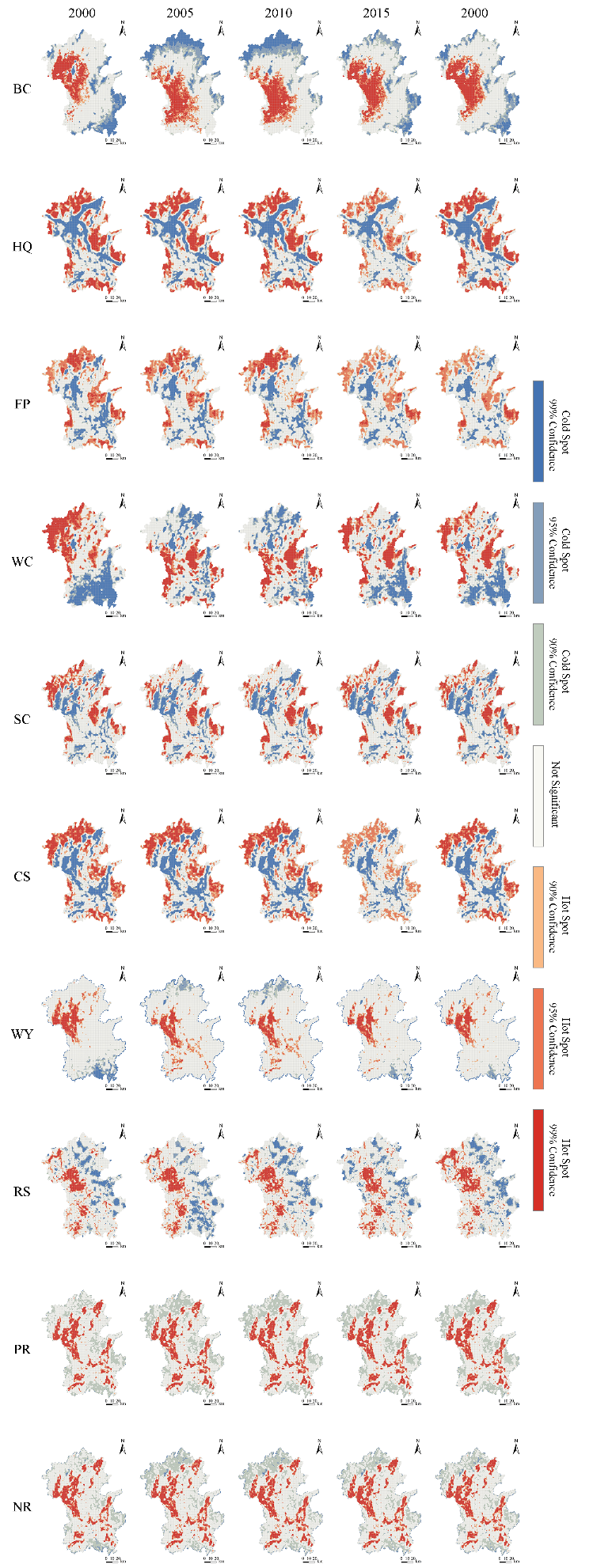


Fig 4. Hot and cold spot distribution of each ecosystem service ( 5 observation years, 10 ecosystem services) (This integrated image corresponds to Figure S2 in the Supplementary Material.)

The subplots in this integrated image were generated using ArcGIS 10.8 (<http://www.esri.com/software/arcgis>), and all subplots were then composited into this integrated image using Adobe Photoshop 2020 (<https://www.adobe.com/cn>). This figure was created independently by the authors.
